# Histidine kinase inhibitors impair shoot regeneration in *Arabidopsis thaliana via* cytokinin signaling and SAM patterning determinants

**DOI:** 10.3389/fpls.2022.894208

**Published:** 2022-09-08

**Authors:** Robin Lardon, Hoang Khai Trinh, Xiangyu Xu, Lam Dai Vu, Brigitte Van De Cotte, Markéta Pernisová, Steffen Vanneste, Ive De Smet, Danny Geelen

**Affiliations:** ^1^HortiCell, Department of Plants and Crops, Faculty of Bioscience Engineering, Ghent University, Ghent, Belgium; ^2^Biotechnology Research and Development Institute, Can Tho University, Can Tho, Vietnam; ^3^Department of Plant Biotechnology and Bioinformatics, Faculty of Sciences, Ghent University, Ghent, Belgium; ^4^Center for Plant Systems Biology, VIB, Ghent, Belgium; ^5^Mendel Centre for Plant Genomics and Proteomics, Central European Institute of Technology (CEITEC), Masaryk University, Brno, Czechia; ^6^Laboratory of Functional Genomics and Proteomics, Faculty of Science, National Centre for Biomolecular Research, Masaryk University, Brno, Czechia; ^7^Lab of Plant Growth Analysis, Ghent University Global Campus, Incheon, South Korea

**Keywords:** phosphoproteomics, kinase inhibitors, cytokinin signaling, shoot regeneration, organogenesis

## Abstract

Reversible protein phosphorylation is a post-translational modification involved in virtually all plant processes, as it mediates protein activity and signal transduction. Here, we probe dynamic protein phosphorylation during *de novo* shoot organogenesis in *Arabidopsis thaliana*. We find that application of three kinase inhibitors in various time intervals has different effects on root explants. Short exposures to the putative histidine (His) kinase inhibitor TCSA during the initial days on shoot induction medium (SIM) are detrimental for regeneration in seven natural accessions. Investigation of cytokinin signaling mutants, as well as reporter lines for hormone responses and shoot markers, suggests that TCSA impedes cytokinin signal transduction *via* AHK3, AHK4, AHP3, and AHP5. A mass spectrometry-based phosphoproteome analysis further reveals profound deregulation of Ser/Thr/Tyr phosphoproteins regulating protein modification, transcription, vesicle trafficking, organ morphogenesis, and cation transport. Among TCSA-responsive factors are prior candidates with a role in shoot apical meristem patterning, such as AGO1, BAM1, PLL5, FIP37, TOP1ALPHA, and RBR1, as well as proteins involved in polar auxin transport (e.g., PIN1) and brassinosteroid signaling (e.g., BIN2). Putative novel regeneration determinants regulated by TCSA include RD2, AT1G52780, PVA11, and AVT1C, while NAIP2, OPS, ARR1, QKY, and aquaporins exhibit differential phospholevels on control SIM. LC–MS/MS data are available *via* ProteomeXchange with identifier PXD030754.

## Introduction

Reversible protein phosphorylation is a post-translational modification (PTM) with a widespread regulatory function in biological systems, as it can alter protein activity, structure, stability, subcellular localization and interactions with other proteins (Park et al., 2012; van Wijk et al., 2014). In plants, phosphorylation is involved in virtually all processes, from growth and development to immunity and abiotic stress resistance, largely due to its role in signal transduction and fine-tuning metabolism (Wang et al., 2007; Park et al., 2012). This is also reflected in the abundance and diversity of kinases and phosphatases in the genome of *Arabidopsis thaliana* (Wang et al., 2007; van Wijk et al., 2014). In particular the receptor-like protein kinases (RLKs), mitogen-activated protein kinase kinase kinases (MKKKs), calcium-dependent protein kinases (CDPKs) and type 2C protein phosphatases (PP2Cs) constitute large families in this model plant (Wang et al., 2007). Phosphate groups can be transferred to the side chains of several amino acids in a peptide, but the most common ones are serine (Ser) and threonine (Thr), followed by tyrosine (Tyr; Park et al., 2012). These are examples of O-phosphorylation, but in rare cases N atoms in histidine (His), lysine (Lys) or arginine (Arg) residues can be phosphorylated as well. Although less common and less stable than phosphoesters, phosphohistidines act as intermediates in two-component signaling relays that are key for environmental adaptation in plants (Ross, 2007). These systems are built around histidine kinases, of which there are at least eight in *Arabidopsis*, including two ethylene receptors (ETHYLENE RESPONSE 1 (ETR1) and ETHYLENE RESPONSE SENSOR 1 (ERS1)), one putative osmosensor (ARABIDOPSIS HISTIDINE KINASE 1 (AHK1)), three cytokinin receptors (AHK2, AHK3 and AHK4/CYTOKININ RESPONSE 1 (CRE1)/WOODEN LEG (WOL)) and two receptors whose function remains unclear (CYTOKININ INSENSITIVE 1 (CKI1) and CKI2/AHK5; Hwang et al., 2002; Nongpiur et al., 2012).

Plant regeneration through *de novo* organogenesis refers to the reconstruction of body parts upon wounding or *in vitro* cultivation, which is key for survival and serves a plethora of biotechnological applications (Ikeuchi et al., 2016, 2019). For example, adventitious roots or shoots can be formed from excised tissue explants for mass clonal propagation and genetic engineering requires regeneration of intact plants from transformed protoplasts (Lardon and Geelen, 2020). These processes rely on plant hormones, such as auxin, cytokinin (CK), brassinosteroids (BR), jasmonic acid (JA), etc. Especially the ratio of auxin to CK determines the identity of regenerating organs, because auxins promote root development and cytokinins stimulate shoot formation (Motte et al., 2014b). Notably, many hormone signal transduction pathways involve phosphorylation. A prime example of this is CK, which is perceived by a two-component His-Asp phosphorelay, that starts with the activation of one of three hybrid receptors (AHK2-4; Schaller et al., 2015; Kieber and Schaller, 2018). Binding of CK to the extracytosolic CHASE domain triggers autophosphorylation of a conserved His residue and transfer of the phosphate to an Asp residue in the receiver domain. From here, it is passed on to ARABIDOPSIS HISTIDINE PHOSPHOTRANSFER (AHP) proteins, that move into the nucleus and activate ARABIDOPSIS RESPONSE REGULATORS (ARRs). B-type ARRs contain a MYB-like DNA-binding domain and induce a transcriptional response, while A-type ARRs induced by the former impose a negative feedback on the signal through competition (Kieber and Schaller, 2018). Several auxin biosynthetic enzymes, PIN efflux carriers and AUXIN RESPONSE FACTORS (ARFs) are regulated by phosphorylation as well (Tan et al., 2021). Moreover, BRs are required for regeneration, as they control organ boundaries in the shoot apical meristem (SAM; Bell et al., 2012; Gendron et al., 2012), they mediate cell division in the root quiescent center (González-García et al., 2011; Lozano-Elena et al., 2018) and biosynthetic mutants show reduced callus growth and shoot induction (Cheon et al., 2010). In the presence of BR, BRASSINOSTEROID INSENSITIVE 1 (BRI1) and BRI1 ASSOCIATED KINASE 1 (BAK1) heterodimerize and initiate a cytoplasmic phosphorylation cascade that causes degradation of BRASSINOSTEROID INSENSITIVE 2 (BIN2; a GSK3/SHAGGY-like kinase) and increased levels of dephosphorylated BRASSINAZOLE RESISTANCE 1 (BZR1) and BRI1-EMS-SUPPRESSOR 1 (BES1). These transcription factors (TFs) induce a transcriptional response that controls cell elongation and division, underscoring the central role of phosphorylation in hormone-regulated regeneration (Cheon et al., 2010; Lozano-Elena et al., 2018).

Besides hormone signaling, phospho-regulation of organogenesis is mediated by specific kinases and phosphatases. For instance, the leucine-rich repeat (LRR)-RLK CLAVATA1 (CLV1) perceives CLV3 in a feedback loop with WUSCHEL (WUS), a TF that determines the balance between proliferation and differentiation in the SAM (Wang et al., 2007; Somssich et al., 2016). CLV1 can be dephosphorylated by KINASE ASSOCIATED PROTEIN PHOSPHATASE (KAPP) and it acts upstream of the PP2C phosphatases POLTERGEIST (POL) and POL-LIKE 1 (PLL1), which are essential for SAM establishment (Yu et al., 2003; Wang et al., 2007; Song et al., 2020). The CLV1 homologs BARELY ANY MERISTEM (BAM) 1–3 also regulate stem cell maintenance in the shoot, but they have broad expression patterns and their function is opposite to that of CLV1 (DeYoung et al., 2006). Other LRR-RLKs that play a role in regeneration include STRUBBELIG (SUB), which controls cell division in the SAM (Chevalier et al., 2005), RECEPTOR-LIKE PROTEIN KINASE 1 (RPK1), a potential abscisic acid receptor that responds to abiotic stresses and underlies natural variation in shoot regeneration from root explants (Motte et al., 2014a) and SOMATIC EMBRYOGENESIS RECEPTOR-LIKE KINASES (SERKs), that are specifically induced during somatic embryogenesis and enhance the process when overexpressed (Méndez-Hernández et al., 2019). Note that BAK1 is a member of the SERK family that also takes part in a developmental pathway encompassing the MKKK YODA (YDA; Wang et al., 2007). In short, SERKs interact with ERECTA (ER) family RLKs and possibly BR signaling kinases [e.g., SHORT SUSPENSOR (SSP) and the BRI1 target BSK1] found downstream or parallel of the ER-SERK complex in the phosphorylation of YDA, which can also occur directly by BIN2 and controls a MAPK cascade composed of MKK4 and MKK5 upstream of MPK3 and MPK6 to mediate embryonic patterning, inflorescence architecture and stomata formation (Kim et al., 2012; Meng et al., 2013; Li et al., 2019; Neu et al., 2019). This cascade is also activated by auxin *via* a non-canonical pathway involving transmembrane kinases (TMKs) 1 and 4 to regulate cell division during lateral rooting (Huang et al., 2019b). MKK7 and PINOID (PID) further control auxin responses by fine-tuning polar transport (Wang et al., 2007). Additionally, the RLK ARABIDOPSIS CRINKLY 4 (ACR4), involved in founder cell specification during lateral root initiation and expressed in the SAM during embryogenesis, has been proposed as a marker for acquisition of organogenic competence (De Smet et al., 2008; Motte et al., 2014b).

In an approach termed chemical genetics, small organic molecules that perturb specific biological processes are used to study a phenotype of interest (Blackwell and Zhao, 2003; Mccourt and Desveaux, 2010). The advantage of this strategy over reverse mutational analysis is that it overcomes functional redundancy and lethality, while enabling temporal and dosage control (Hicks and Raikhel, 2012, 2014; Xuan et al., 2013). Disadvantages, however, include possible off-target effects, poor uptake, and metabolic conversion. In the context of phosphorylation, several compounds have been used to block the function of protein kinases. Examples of such kinase inhibitors are rapamycin, AZD8055, bikinin, olomoucine, 3,3′,4′,5-tetrachlorosalicylanilide (TCSA) and Closantel® (Papon et al., 2003; De Rybel et al., 2009; Sheremet et al., 2010; Van Leene et al., 2019). The TARGET OF RAPAMYCIN (TOR) is an evolutionary conserved Ser/Thr kinase that balances growth in response to nutrient availability and environmental stresses by modulation of protein synthesis (Ren et al., 2012; McCready et al., 2020). It is also involved in embryo development, meristem activation, auxin signaling, adventitious rooting and DNA methylation (Menand et al., 2002; Xiong et al., 2013; Deng et al., 2016, 2017; Zhu et al., 2020a). Notably, rapamycin depends on FKBP12 to block TOR function and reduced affinity for the plant FKBP12 homolog causes insensitivity of *Arabidopsis* seedlings to physiological concentrations of the compound (Menand et al., 2002; Ren et al., 2012). Nonetheless, hypoxia partially restores inhibition and there is substantial overlap in the phosphoproteome of cell cultures treated with rapamycin or the potent TOR inhibitor AZD8055 (Deng et al., 2016; Van Leene et al., 2019). Rapamycin also blocks glucose-induced root growth *via* the TOR kinase (Xiong et al., 2013). Bikinin inhibits GSK3-like kinases (e.g., BIN2; De Rybel et al., 2009) and olomoucine targets cyclin-dependent protein kinases, thereby blocking cell cycle progression and altering root morphology (Sheremet et al., 2010). TCSA and Closantel® inhibit bacterial histidine kinases and impede CK signaling in periwinkle cell cultures (Papon et al., 2003). In the same system, these drugs hamper ethylene-induced alkaloid biosynthesis *via* ETR1 (Papon et al., 2004). In *Arabidopsis*, sensing of H_2_O_2_ by AHK5 to integrate endogenous and environmental stimuli (e.g., darkness, ethylene, and nitric oxide) in stomatal guard regulation was blocked by TCSA (Desikan et al., 2008). Other phenotypic effects of TCSA and Closantel® in plants have not been investigated. These examples illustrate that Closantel® and TCSA target His kinase activities in plants. However, salicylanilide derivatives disrupt membrane integrity and long-term exposure causes toxic effects on cell growth (Papon et al., 2003).

Despite the abundance of evidence suggesting that regeneration depends on phosphorylation, comprehensive studies of phosphoproteome dynamics have been restricted to protoplast regeneration in moss and cell dedifferentiation in *Arabidopsis* (Chitteti and Peng, 2007; Wang et al., 2014b). This uncovered a variety of phosphopeptides involved in cell wall metabolism, cytoskeleton structure, signal transduction, transcriptional regulation, cell division and photosynthesis. Here, we explored the effect of three kinase inhibitors on shoot regeneration from root explants in *Arabidopsis thaliana* and optimized the application of TCSA for downstream analyses. We then examined how this compound interacts with cytokinin signaling by testing mutants and reporter lines. Finally, we exploited mass spectrometry (MS)-based shotgun proteomics to probe phosphoproteome changes upon TCSA treatment and identify phosphorylation events that govern *de novo* shoot organogenesis parallel or downstream of cytokinin perception.

## Materials and methods

### Plant materials and chemicals

Natural *Arabidopsis thaliana* (L.) Heynh. accessions Lp2-2, Kyoto, Kz-9, Sg-1, and Db-1 were obtained from the Nottingham *Arabidopsis* Stock Centre (NASC; N76636). Ws and Col-0 are descendants from seed stocks purchased at the Versailles *Arabidopsis* Stock Center (INRA; 530AV) and NASC (N76778). Cytokinin mutants *ahk2-5*, *cre1 − 2*, *ahk2-5 ahk3-7*, *ahk2-5 cre1-2*, *ahk3-7 cre1-2*, *ahk2-5 ahk3-7/AHK3 cre1-2* (Riefler et al., 2006), *ahk2 − 1*, *ahk3-1* (Nishimura et al., 2004; Jeon et al., 2010), *ahk2-2 ahk3-3* (Higuchi et al., 2004; Jeon and Kim, 2013), *ahp2-1* (CS860144), *ahp3* (CS660145), *ahp5-2* (CS860148), *ahp2-1 ahp3* (CS860151), *ahp2-1 ahp5-2* (CS860153), *ahp3 ahp5-2* (CS860155), *ahp2-1 ahp3 ahp5-2* (CS860161), *arr1-3* (CS6971; Jeon and Kim, 2013), *35S:ARR1-SRDX* (Heyl et al., 2008), and ARR1 phosphomimic (*35S:ARR1^D94E^ arr1-1*; Kurepa et al., 2014a) are all in Col−0 background and were described previously. Reporter lines carrying *proTCSn::GFP* and *proWUS::tdTom* in WT Col−0 and *ahk4* backgrounds were kindly provided by Markéta Pernisová (Masaryk University; Pernisova et al., 2018). *ProDR5::GFP* and *proSTM::GFP* were obtained from NASC (respectively N9361 and N68954).

Rapamycin and TCSA (3,3′,4′,5-Tetrachlorosalicylanilide) were purchased from Fisher Scientific™ (respectively Catalog No. 15434849; CAS No. 53123–88-9, Catalog No. 10473741; CAS No. 1154-59 − 2) and Closantel® (5′-Chloro-4′-(4-chloro-α-cyanobenzyl)-3,5-diiodo-2′-methylsalicylanilide, *N*-[5-Chloro-4-(4-chloro-α-cyanobenzyl)-2-methylphenyl]-2-hydroxy-3,5-diiodobenzamide) was obtained from Sigma-Aldrich (Product No. 34093; CAS No. 57808–65-8). Kinase inhibitors were dissolved in DMSO (dimethyl sulfoxide) at a stock concentration of 10 mM and filter sterilized before addition to the SIM.

### Regeneration assays

The protocol for shoot regeneration from root explants was adapted from the work of Valvekens et al. (1988). Seeds were sterilized by exposure to chlorine gas for 4 h and sown on Gamborg B5 medium [3.1 g/l B5 salts including vitamins, 0.05% 2-(4-morpholino)-ethane sulfonic acid (MES), 2% (w/v) glucose and 0.7% agar at pH 5.8]. After vernalization at 5°C for 4 days, seedlings were grown under warm white fluorescent tungsten tubes (~100 μM m^−2^ s^−1^) at 21°C following a 14/10 h light/dark regime for 10 days. Next, 7 mm long root segments including the tip were excised and placed on CIM [B5 supplemented with 2.2 μM 2,4-dichlorophenoxy acetic acid (2,4-D) and 0.2 μM kinetin] for 4 days. Finally, the explants were transferred to SIM [B5 supplemented with 5 μM 2-isopentenyl adenine (2-IP) and 0.86 μM 3-indole acetic acid (IAA)] and incubated for 21 days. Timed application of kinase inhibitors at this stage was achieved by transferring explants to new SIM plates supplemented with 10 μM rapamycin, TCSA or Closantel® for the indicated periods [the concentration is based on experiments by Desikan et al., 2008]. Untreated controls were kept on the original plate. To score regeneration, pictures were taken using binoculars (6.3×) and the green area was determined in ImageJ. Further data processing and plot construction was done in R.

### Microscopy

Root explants were subjected to the regeneration protocol described above and harvested right before transfer to SIM (4dCIM), after 24 h on SIM (24hSIM), and after 28, 48, 72, and 120 h on SIM (28hSIM, 48hSIM, 72hSIM, and 120hSIM) following incubation on SIM supplemented with 10 μM TCSA or an equal amount of DMSO between 24 and 28 h (24 h-28h_TCSA and 24 h-28h_mock). Slides were prepared with 150 μl of Milli-Q water and for each combination of reporter line, treatment, and time, 6–12 explants were analyzed (1–6 non-overlapping segments were recorded per explant). Imaging was done at 10× in CellSens using an Olympus IX81 fluorescence microscope powered by an X-cite® 120 LED Boost lamp coupled to an Olympus XM10. Exposures were manually set to 150 ms for *proTCSn::GFP* lines, 1 s for *proWUS::tdTom* lines and 500 ms for *proDR5::GFP* and *proSTM::GFP* lines. For GFP measurements, a U-FCFP Fluorescence Filter Cube (BX3) was used (excitation filter: BP 425–445 nm, dichromatic mirror: DM 455 nm and emission filter: BP 460–510 nm) and for tdTom detection, a U-MWU2 Fluorescence Filter Cube (BX2/IX2) was applied (excitation filter: BP 330–385 nm, dichromatic mirror: DM 400 nm and emission filter: LP 420 nm). Corrected total cell fluorescence was determined in ImageJ by auto thresholding the image (using the “triangle white” method) to select the explant and then subtracting the mean background intensity multiplied by the explant area from the integrated density of pixels within the explant.

### Phosphoproteomics

Per sample, 200–300 seedlings were subjected to the shoot regeneration protocol described above. Instead of sampling individual root explants, however, plantlets were grown in a horizontal line and the lower 2 cm of all root systems was cut with a razor for transfer between media. Harvesting was done by flash freezing ~250 mg of roots in liquid nitrogen before grinding in a Retch® mill for 1 min at 20 Hz, followed by 30 s at 30 Hz. Protein extraction, digestion, phosphopeptide enrichment and LC–MS/MS analysis were carried out according to Vu et al. (2016). First, Tris–HCl homogenization buffer (containing 50 mM Tris–HCl, 0.1 M KCl, 5 mM EDTA, 500 mM DTT, and 30% sucrose at pH 8.0), supplemented with 1 Protease Inhibitor Cocktail Tablet (Roche) and 1 PhosSTOP Phosphatase Inhibitor Cocktail Tablet (Roche) per 50 ml, was added to the samples. Debris was removed by sonication and centrifugation at 4°C and 2,500 g for 15 min. Next, proteins were precipitated by chloroform extraction and centrifuged, after which 20 ml methanol was added to mix with the aqueous phase in each tube. After another centrifugation, pellets were washed with 80% acetone and resuspended in 50 mM triethylammonium bicarbonate (TEAB) buffer containing 6 M guanidinium hydrochloride (pH 8.0). Cysteine alkylation was done by addition of Tris-(2-carboxyethyl) phosphine (TCEP, Pierce) and iodoacetamide (Sigma-Aldrich) in final concentrations of 15 and 30 mM, respectively, and the reaction was allowed to proceed for 15 min at 30°C (in the dark). Next, 2.5 mg of each sample was pre-digested using 1 aliquot of 10 μg EndoLysC (Wako), while mixing for 2.5 h (in the dark). Samples were then diluted 8× in 50 mM TEAB, followed by overnight digestion with trypsin (Promega Trypsin Gold; mass spectrometry grade). Both digestions occurred at 37°C using an enzyme-to-substrate ratio of 1% w/w. To stop digestion, samples were acidified to pH ≤ 3.0 with TFA, before desalination using SampliQ C18 SPE cartridges (Agilent) according to the manufacturer’s guidelines. Subsequently, the digests were vacuum-dried and redissolved in loading solvent for enrichment of phosphopeptides using Ti-IMAC beads. After elution, samples were acidified, vacuum-dried and resuspended in acetonitrile solution for liquid chromatography on an Ultimate 3000 RSLC nano LC (Thermo Fisher Scientific). This was coupled in-line to a Q Exactive mass spectrometer (Thermo Fisher Scientific) operated in data-dependent, positive ionization mode and using HCD collision to acquire MS/MS scans for the 10 most abundant precursor ions in each MS1 spectrum (Vu et al., 2016). The mass spectrometry proteomics data have been deposited to the ProteomeXchange Consortium *via* the PRIDE (Perez-Riverol et al., 2019) partner repository with the dataset identifier PXD030754.

Identification and quantification of (phospho) peptides from MS/MS spectra was done in MaxQuant (version 1.6.11.0; Supplementary Data 2; Cox and Mann, 2008; Tyanova et al., 2016a). Methionine oxidation, protein N-terminal acetylation and phosphorylation of S, T, and Y residues were chosen as variable modifications, and cysteine carbamidomethylation was included as fixed modification (with maximum five modifications per peptide). Trypsin was selected for digestion, allowing cleavage at lysine or arginine residues followed by proline, and up to 2 missed cleavages. Respective precursor mass tolerances of 20 and 4.5 ppm were used for the first and main search, intensity thresholds for MS1 and MS2 were set to 500, and DIA quantification and feature quantification methods were, respectively set to “Top fragments by annotation” and “Scan” (using the top three fragments and a top MS/MS intensity quantile of 0.8). DIA mass window factor, background subtraction quantile and factor were all set to 0, and DIA XGBoost sub sample was set to 0.65, with a binary logistic learning objective and minimum child weight of 3. Default values (in ppm) were used for other instrument settings. Database searches were done against a FASTA file with protein sequences for all predicted transcripts in *Arabidopsis thaliana* Ws-0, derived from the 19 Genomes Project (Gan et al., 2011). Decoy mode was kept at “Revert” and default parameters were used under the protein quantification, label-free quantification, and MS/MS analyzer tabs. An FDR filter of 1% was applied to peptide spectrum matches, protein identifications, and modification sites, and the “Match between runs” feature was used (with a match time window of 0.7 min, match ion mobility window of 0.05, alignment time window of 20 min, and alignment ion mobility of 1). The Phospho (STY)Sites.txt table produced by MaxQuant was uploaded in Perseus (version 1.6.10.50; Tyanova et al., 2016b) to remove potential contaminants and reverse hits. Also, rows were filtered to have a localization probability ≥ 0.75, site multiplicities were expanded, and intensity values were log_2_ transformed. Next, the data were uploaded in R for statistical analysis with the DEqMS package (see Supplementary Code; results are available in Supplementary Data 1; Zhu et al., 2020b).

### Gene set enrichment analysis

GSEA was performed with the ClueGO and CluePedia plugins for Cytoscape (Shannon et al., 2003; Bindea et al., 2009, 2013). All 522 genes corresponding to significantly deregulated phosphosites between TCSA and mock treatment on SIM (FDR ≤ 0.05) were uploaded in ClueGO and compared to a predefined reference set containing 25,386 Ensembl gene identifiers for *Arabidopsis thaliana* using a right-sided hypergeometric test and correcting for multiple tests according to Benjamini-Hochberg. Enriched GO Biological Process and Immune System Process terms (Ashburner et al., 2000; The Gene Ontology Consortium, 2014), KEGG pathways (Kanehisa and Goto, 2000), PO Anatomy—Plant Structure, and PO Temporal—Plant Growth and Developmental Stage terms (Avraham et al., 2008) with FDR ≤ 0.03, 5 ≤ GO tree interval level ≤ 12, ≥3 associated genes, and ≥5% associated genes were retained (using all evidence codes). GO term fusion and grouping options were enabled, “Groups” was selected under visual style, and the connectivity threshold (kappa score) was set to 0.3. Grouping was based on kappa scores, using random colors, an initial group size of 1, and group merge percentages of 50% (terms/genes). Overview terms were defined by highest significance. The yFiles Organic Layout was used for network construction, and only genes from the input list mapping to enriched functional terms were included in the graph. The resulting network contains 75 functional terms (connected by 103 edges and divided into 16 groups), 194 genes, and 838 edges. Centrality parameters in the network (degree and betweenness) were calculated with CentiScaPe (Scardoni et al., 2009). A bar plot reflecting less stringent settings (FDR ≤ 0.05, 5 ≤ GO tree interval level ≤ 15, ≥2 associated genes, and ≥5% associated genes) is presented in Supplementary Figure 2, including 115 functional terms divided into 26 groups.

### Statistics and reproducibility

All experiments were performed once. In the regeneration assays, 24–30 explants were analyzed per combination of treatment and line, and 6–12 root segments were harvested for microscopic analysis. As the calculated green area and corrected total cell fluorescence were not normally distributed, non-parametric statistics were used. Global significance was assessed by Kruskal–Wallis tests and pairwise comparisons were based on Dunn’s tests or Wilcoxon rank sum tests (as explained in the figure captions). For the phosphoproteome analysis, five independent biological replicates were sampled for each of the three design points and two technical replicates were performed in each LC–MS/MS run. Only phosphosites detected in ≥2 samples for each treatment group were retained in the statistical analysis, and run effects were normalized by subtracting the median log_2_ sample intensity. For DEqMS analysis, the minimum number of unique and razor peptides used for quantification of each phosphosite across samples was retrieved from the proteinGroups.txt table, and limma (Ritchie et al., 2015) was run in robust trend mode. Volcano plots and heatmaps were, respectively constructed with the EnhancedVolcano and ComplexHeatmap packages in R (Gu et al., 2016).

## Results

### Kinase inhibitors modulate shoot regeneration

**Figure 1:**
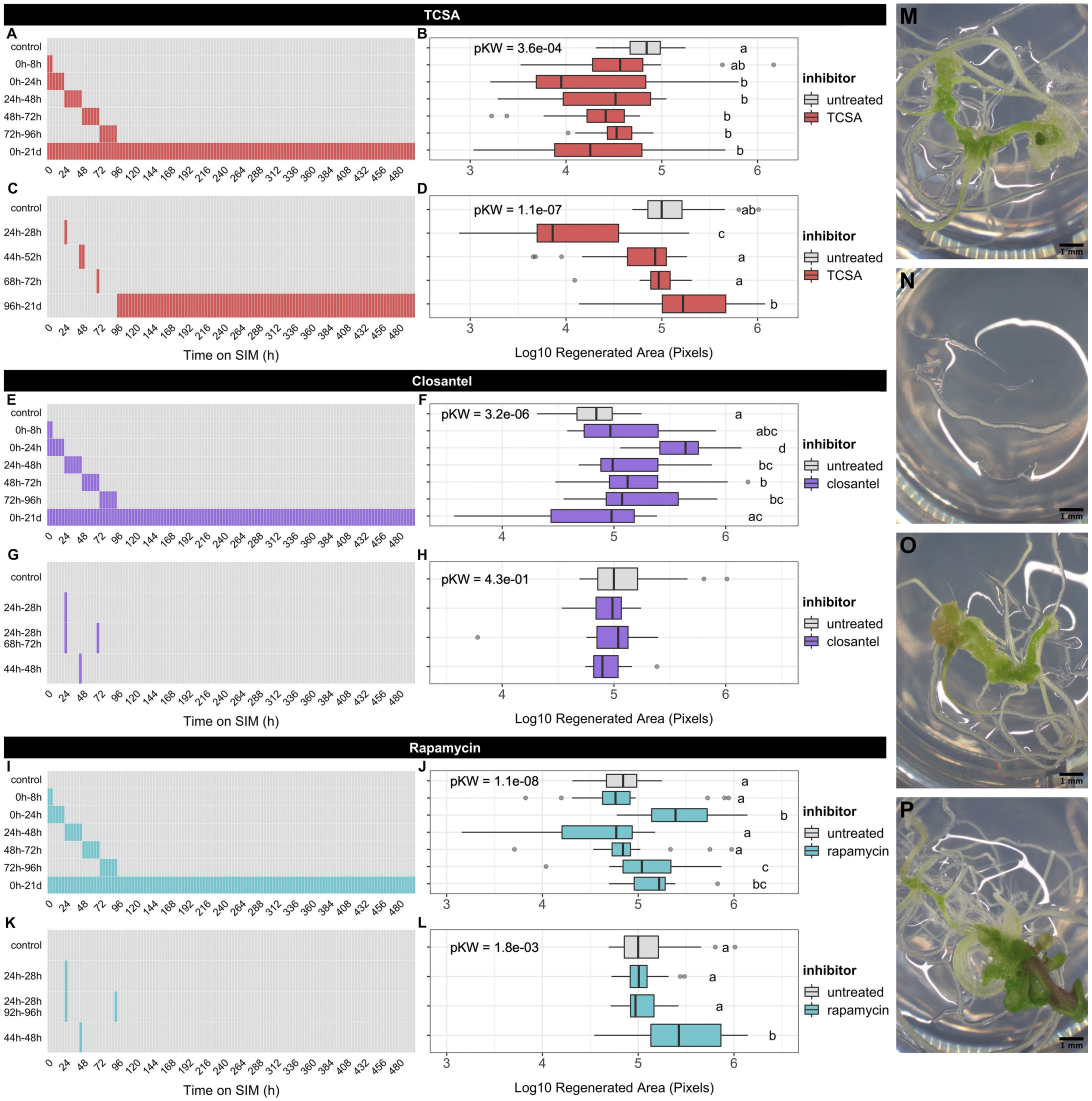
Effect of kinase inhibitors on shoot regeneration from root explants of *Arabidopsis thaliana* ecotype Ws. (**A,C**) Timetable of broad and narrow TCSA application windows on SIM. (**B,D**) Regenerated area after TCSA treatment following the schemes in (**A,C**). (**E,G**) Timetable of broad and narrow Closantel® application windows on SIM. (**F,H**) Regenerated area after Closantel® treatment following the schemes in (**E,G**). (**I,K**) Timetable of broad and narrow rapamycin application windows on SIM. (**J,L**) Regenerated area after rapamycin treatment following the schemes in (**I,K**). Red, purple, and aquamarine boxes, respectively indicate SIM supplemented with 10 μM TCSA, Closantel® or rapamycin, while gray boxes represent control SIM. Boxplots depict the log_10_-transformed area of green tissue (in pixels) regenerated from roots after 21 days on SIM. Per combination of inhibitor and application window, ~24 individual explants were analyzed. Global significance was assessed using Kruskal Wallis tests per panel and shown as FDR-adjusted *p*-values (pKW) in each plot. Pairwise comparisons are based on Dunn’s non-parametric tests and shown as a compact letter display (FDR ≤ 0.05). (**M–P**) Representative images of Ws root explants after 21 days on control SIM (**M**) or TCSA treatment from 24 to 28 h (**N**), Closantel® treatment from 24 to 28 h (**O**), and rapamycin treatment from 44 h to 48 h (**P**). Pictures were taken using binoculars (6.3×) and black scale bars represent 1 mm.

Given the pivotal role of cytokinin perception and protein phosphorylation in *de novo* shoot organogenesis, we sought to delineate the time window of critical phosphorylation events for regeneration by interfering with histidine kinases using TCSA and Closantel® (Papon et al., 2003, 2004). As a negative control, we included rapamycin, which has limited impact on plant development under physiological conditions because of structural divergence in the FKBP12 protein required to block TOR kinase activity (Menand et al., 2002; Li et al., 2012). Root explants of *Arabidopsis thaliana* (ecotype Wassilewskija; Ws) were subjected to a two-step protocol for shoot regeneration, in which they are pre-incubated on auxin-rich callus-induction medium (CIM) for 4 days, before transfer to cytokinin-rich shoot induction medium (SIM). After 21 days on SIM, the regeneration rate was scored by measuring the green area of explants. Initially, the impact of kinase inhibitors on shoot induction was assessed using broad application intervals: from 0 to 8 h, 0 to 24 h, 24 to 48 h, 48 to 72 h, 72 to 96 h, or during all 21 days on SIM (Figures 1A,B,E,F,I,J). Subsequently, we tested narrower treatment windows for each inhibitor, based on the results of the first screen (Figures 1C,D,G,H,K,L). Chemicals were applied at a concentration of 10 μM based on previous reports (Papon et al., 2003; Desikan et al., 2008) and an experiment showing that 10 μM TCSA yields the most specific reduction of regeneration in Ws, while balancing toxic off-target effects (Supplementary Figure 1).

**Figure 2:**
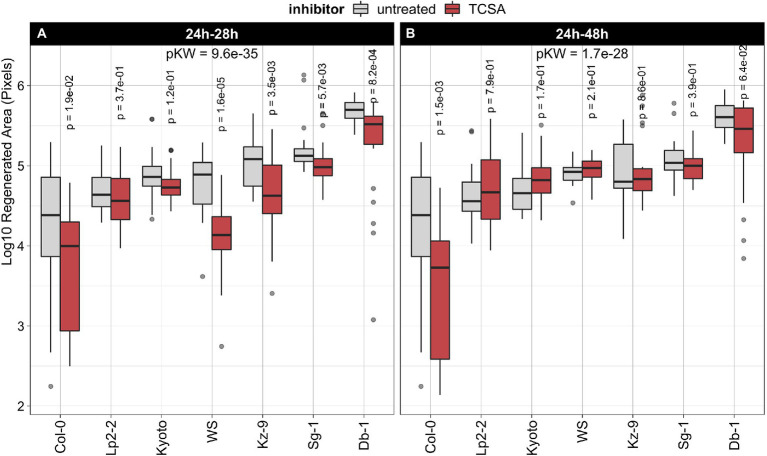
Effect of TCSA on regeneration in seven natural Arabidopsis thaliana accessions. (**A**) Treatment with 10 μM TCSA from 24 to 28 h on SIM. (**B**) Treatment with 10 μM TCSA from 24 to 48 h on SIM. Boxplots show the log_10_-transformed area of green tissue (in pixels) regenerated from root explants after 21 days of SIM incubation. Red boxes indicate TCSA treatment and gray boxes reflect control measurements. Per combination of accession and application window, ~24 individual explants were analyzed. FDR-adjusted *p*-values from Kruskal Wallis tests (pKW) express global significance in each panel and *p*-values for TCSA treatment within each accession are deduced from pairwise Wilcoxon’s tests using the untreated controls as reference.

We find that TCSA is detrimental to shoot regeneration, while Closantel® and rapamycin either have no effect or promote the process, depending on the time of application (Figure 1). Intriguingly, TCSA is most potent when applied during the first 4 days of SIM incubation, especially when applied in short intervals, as exposure from 0 to 24 h and 24 to 28 h yields the lowest regeneration rates and treatment from day 5 to day 21 on SIM even has a slightly positive influence (Figures 1A–D,M,N). An inverse trend is observed for Closantel®, which has no significant effects when applied for less than 24 h (Figures 1G,H,O) and promotes shoot regeneration when administered during the first 4 days of SIM incubation. The latter effect is strongest from 0 to 24 h (Figures 1E,F). Contrary to the reported lack of effects of rapamycin on vegetative growth (Menand et al., 2002), this compound enhances *in vitro* shoot formation in our experiments. This effect occurs at later application windows than observed for Closantel® (e.g., 44 to 48 h, 72 to 96 h or 0 to 21 days on SIM; Figures 1I–L,P), and hints at involvement of rapamycin-sensitive Ser/Thr kinases that negatively regulate *de novo* organogenesis. Note that rapamycin also promotes regeneration when administered in the initial 24 h. Overall, root explants are most susceptible to TCSA and Closantel® from 8 to 28 h on SIM, as the 0 to 8 h treatment does not have substantial effects on shoot regeneration. In particular, the data indicate that shoot regeneration is highly sensitive to a pulse of TCSA administered from 24 to 28 h on SIM, so this treatment was studied in subsequent analyses.

### Regeneration in natural accessions is differentially reduced by TCSA

Next, we assessed the robustness of TCSA effects by comparing regeneration rates in seven natural *Arabidopsis thaliana* accessions after treatment with this inhibitor, using the same setup as before. We selected Columbia-0 (Col-0) and Wassilewskija (Ws) because they are commonly used genetic backgrounds and included five other accessions previously shown to have a high capacity for shoot regeneration (Lardon et al., 2020). Evaluating untreated explants of these strains after 21 days on control SIM shows that Col-0 regenerates poorly, while Ws and the other ecotypes are average to strong performers. For an optimal inhibitory effect on shoot organogenesis (Figures 1A–D), TCSA was applied to root explants from 24 to 28 h on SIM. In all accessions, regeneration is reduced by this pulse TCSA treatment, albeit with variable efficiency (Figure 2A). The absolute difference in regenerated area between control and TCSA-treated explants is largest in the best regenerating ecotypes (e.g., Db-1), but log-fold changes are higher in strains with average regeneration capacities (e.g., Ws). To verify the efficacy of the short pulse treatment across multiple accessions, TCSA was also applied during an extended interval from 24 to 48 h on SIM. While Col-0 explants show increased susceptibility to prolonged TCSA exposure, this treatment has little effect in accessions with a higher regenerative potential (Figure 2B). In conclusion, TCSA treatment from 24 to 28 h on SIM robustly inhibits shoot regeneration in various *Arabidopsis* ecotypes.

### TCSA interferes with cytokinin signaling

**Figure 3:**
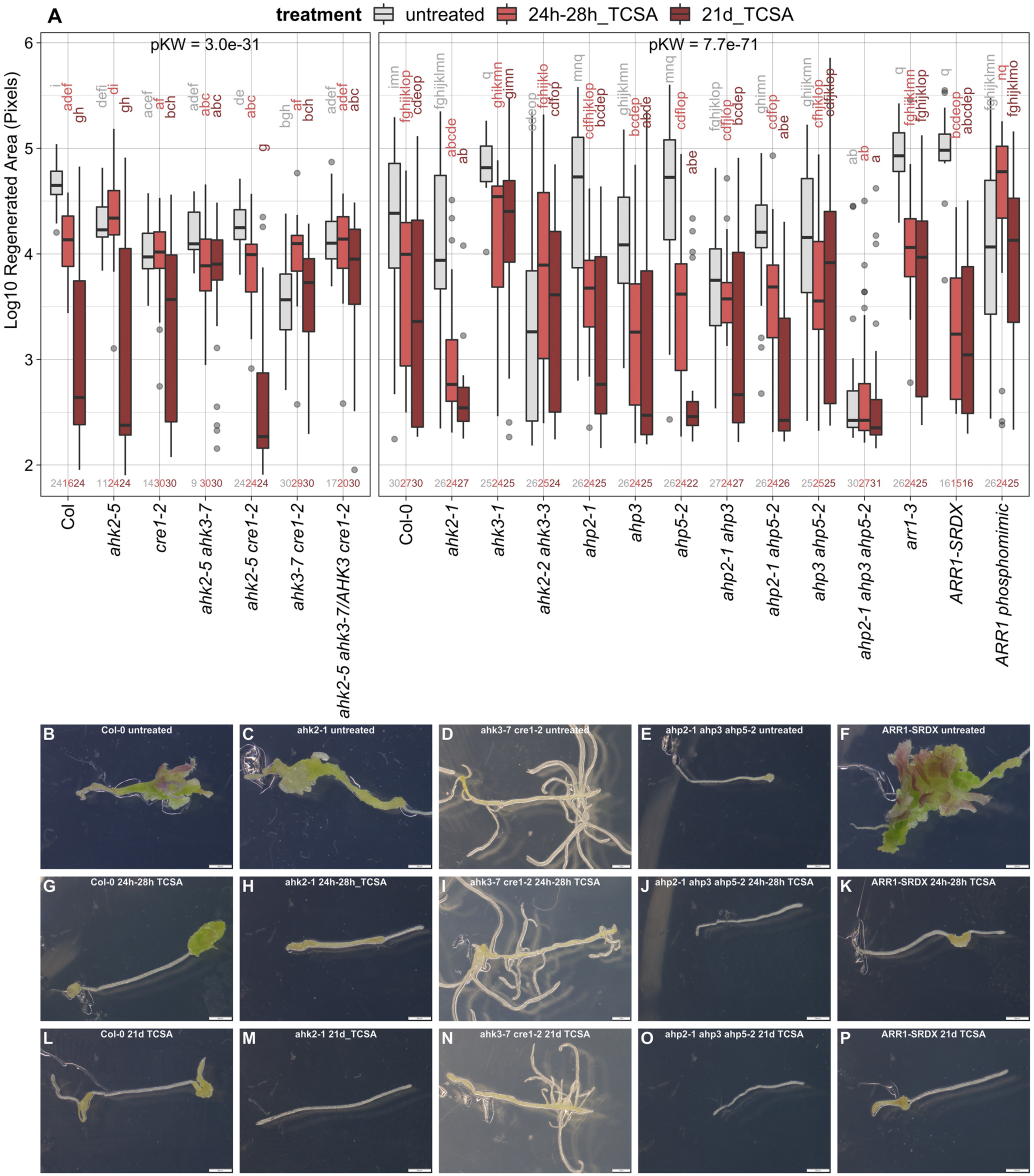
Response of *ahk*, *ahp*, and *arr1* mutants to TCSA. (**A**) Boxplot showing the log_10_-transformed area of green tissue (in pixels) regenerated from root explants incubated on control SIM for 21 days (untreated; gray) or exposed to 10 μM TCSA from 24 to 28 h (bright red) or during the entire incubation period (dark red). Replicate numbers are indicated above the x-axis (generally *n* ≥ 24 explants). Global significance was evaluated by Kruskal Wallis tests (pKW) per panel and pairwise comparisons are shown as a compact letter display based on Dunn’s non-parametric test (FDR ≤ 0.05). (**B–P**) Representative images of WT Col-0 (**B,G,L**), *ahk2-1* (**C,H,M**), *ahk3-7* *cre1-2* (**D,I,N**), *ahp2-1 ahp3 ahp5-2* (**E,J,O**), and *ARR1-SRDX* (*F,K,P*) explants after 21 days on control SIM (**B–F**) or including TCSA application from 24 to 28 h (**G–K**) and from day 0 to day 21 (**L–P**). Pictures were taken using binoculars (6.3×) and white scale bars represent 500 μm.

As the cytokinin signaling machinery operates *via* a His-Asp phosphorelay, it is a prime target for histidine kinase inhibitors in plants. To determine if TCSA affects shoot regeneration by specifically interfering with CK signaling, genetic interaction of TCSA with canonical CK receptors (*AHKs*) and downstream components (*AHPs* and *ARRs*) was evaluated. Hereto, we applied TCSA from 24 to 28 h on SIM, and during the full 21-day incubation period (because most of the available mutants are in Col-0 background and this accession is susceptible to prolonged TCSA treatment; Figure 2). The area of regenerated shoot tissues in wild-type (WT) Col-0 explants declines progressively when extending the exposure to TCSA (Figures 3A,B,G,L). This trend is also apparent in most of the cytokinin mutants, but the magnitude of change is variable. Of the single CK receptor mutants, *ahk2-1* and *ahk2-5* are sensitive to TCSA, while *ahk3-1* and *cre1-2* (*ahk4*) are resistant, suggesting that reduced regeneration by TCSA involves AHK3 and AHK4 (Figures 3A,C,D,H,I,M,N). Consistently, *ahk2-5 ahk3-7*, *ahk2-2 ahk3-3*, *ahk3-7 cre1-2* and the triple mutant are TCSA-resistant as well. Stronger TCSA responsivity in *ahk2-5 cre1-2* compared to *ahk2-5 ahk3-7* or *ahk2-2 ahk3-3* implies that AHK3 is most susceptible to inhibition, although AHK4 was previously reported to be the most important CK receptor for *de novo* organogenesis in roots (Pernisova et al., 2018). Accordingly, regeneration is compromised in untreated *cre1-2* and higher order *ahk* mutants (Figures 3A,D), highlighting the importance of AHK4 (combined with AHK2 and AHK3) for shoot regeneration. Overall, genetic loss of *AHK* function shows interplay with chemical inhibition.

**Figure 4:**
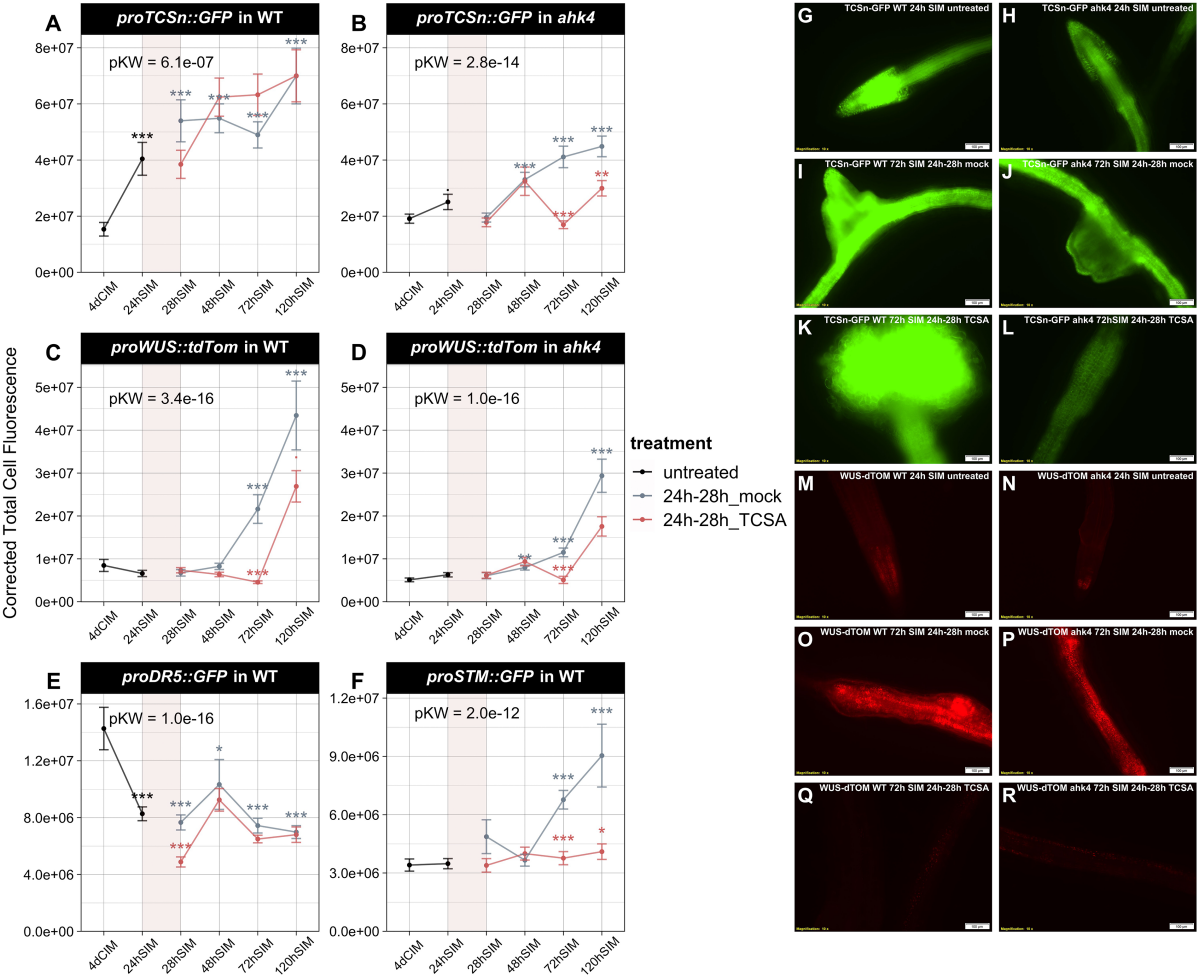
Analysis of TCSn, DR5, WUS, and STM reporters in wild type (WT) and *ahk4* mutants before and after TCSA or mock treatment. (**A,B**) *ProTCSn::GFP* fluorescence in WT and *ahk4*. (**C,D**) *ProWUS::tdTom* fluorescence in WT and *ahk4*. (**E,F**) *ProDR5::GFP* and *proSTM::GFP* fluorescence in WT. Line plots show corrected total cell fluorescence (see Materials and methods) in root explants at several time points before and after treatment with 10 μM TCSA or mock from 24 to 28 h on SIM (represented as a transparent red block in each graph). Global significance was determined by Kruskal Wallis tests per panel (pKW) and asterisks reflect FDR-adjusted *p*-values from pairwise Wilcoxon’s tests comparing TCSA treatment to corresponding mock samples and mock treatment to untreated samples after 4 days on CIM (. = *p* < 0.1, * = *p* < 0.05, ** = *p* < 0.01 and *** = *p* < 0.001). (**G–L**) Representative images of *proTCSn::GFP* fluorescence in WT (**G,I,K**) and *ahk4* (**H,J,L**) after 24 h (**G,H**) and 72 h on SIM with mock (**I,J**) or TCSA treatment (**K,L**) between 24 and 28 h. (**M–R**) Representative images of *proWUS::tdTom* fluorescence in WT (**M,O,Q**) and *ahk4* (**N,P,R**) after 24 h (**M,N**) and 72 h on SIM with mock (**O,P**) or TCSA treatment (**Q,R**) between 24 and 28 h. Pictures were taken using a fluorescence microscope (10×).

Single mutants in phosphotransfer proteins acting downstream of AHKs in CK perception show similar regeneration levels and TCSA responsivity to WT Col-0 (Figure 3A), indicating functional redundance among the AHPs. Whereas *ahp2-1 ahp5-2* and to a lesser extent *ahp2-1 ahp3* remain sensitive to TCSA, *ahp3 ahp5-2* and especially *ahp2-1 ahp3 ahp5-2* are TCSA-resistant. This suggests that AHP3 and AHP5 are susceptible to TCSA. Besides, control regeneration is reduced in *ahp2*-*1 ahp3* and the triple mutant fails to regenerate under all conditions (Figures 3A,E,J,O). Therefore, multiple loss of AHP function phenocopies TCSA treatment, suggesting that AHPs are also involved in TCSA-reduced regeneration. Further downstream of the AHK-AHP signaling cascade, B-type response regulators such as ARR1 activate transcriptional CK responses. ARR1 directly binds the promoter of the shoot stem cell regulator *WUS* (Meng et al., 2017; Zhang et al., 2017), but indirectly represses *CLV3* and *WUS via* competition with ARR12 and induction of the auxin signaling repressor *INDOLE-3-ACETIC ACID INDUCIBLE 17* (*IAA17*; Liu et al., 2020). In agreement with an inhibitory role for ARR1 in shoot development, regeneration is increased in *arr1-3* mutants and explants containing the dominant *ARR1-SRDX* repressor (Figures 3A,F). Congruently, a constitutively active ARR1 phosphomimic (*35S::ARR1^D94E^*) reduces organogenic competence. Sensitivity of *arr1* loss-of-function mutants to TCSA (Figures 3A,F,K,P) implies that ARR1 is not a direct target and corroborates the involvement of upstream signaling modules. Additionally, gain-of-function mutants show reduced control regeneration and TCSA responsivity, which suggests that blocking CK perception has no additional effect over excessive ARR1 activity or vice versa. Taken together, these data confirm that canonical cytokinin signaling is required for shoot formation on SIM and suggest that TCSA inhibits regeneration through inhibition of this pathway.

To elucidate the impact of TCSA on hormone responses during shoot regeneration, we analyzed reporter lines for auxin (*proDR5::GFP*) and cytokinin activity (*proTCSn::GFP*) before and after TCSA or mock treatment from 24 to 28 h on SIM. For comparison, we also assessed transcriptional dynamics of the shoot markers *WUS* and *SHOOT MERISTEMLESS* (*STM*) *via proWUS::tdTom* and *proSTM::GFP*. Because AHK4 is the most important CK receptor for root-to-shoot conversion (Pernisova et al., 2018) and *cre1-2* (*ahk4*) mutants showed differential TCSA responses (Figure 3A), we analyzed *proTCSn::GFP* and *proWUS::tdTom* reporters in WT and *ahk4*. While *proTCSn::GFP* activity in WT steadily increases in the first 5 days upon transfer to cytokinin-rich SIM, it does not reach the same levels in mock-treated *ahk4* explants, confirming the importance of AHK4 for CK perception in the root. Surprisingly, TCSA has limited impact on *proTCSn::GFP* intensity in WT and only represses CK responses in the *ahk4* mutant from day three to day five on SIM (Figures 4A,B,G–L), which suggests that residual CK signals, transmitted by TSCA-resistant AHKs during SIM incubation, are sufficient to trigger the sensitive *proTCSn::GFP* reporter. Similarly, *proWUS::tdTom* intensity in WT rises after 3 days on SIM and this induction is delayed by TCSA application (Figures 4C,M,O,Q). In *ahk4*, *WUS* transcription is also induced from day three to day five, but it only reaches about 70% of WT expression in the mock condition and the response is even lower after administering TCSA (Figures 4D,N,P,R). Despite the limited *proTCSn::GFP* activity in this context, there is a slight surge in *WUS* levels in TCSA-treated *ahk4* roots, indicating residual CK responses or activation of *WUS* transcription by other pathways. The *proDR5::GFP* marker is rapidly repressed after transfer to SIM in both mock and TCSA-treated root segments (Figure 4E), which shows that auxin is not a primary determinant of TCSA-impaired shoot regeneration. On the other hand, *proSTM::GFP* intensity strongly increases after 2 days on SIM in the mock condition, but this effect is not observed after exposure to TCSA (Figure 4F). Hence, STM is a downstream target of TCSA, acting in parallel of reduced AHK signaling and delayed *WUS* expression. Deregulation of shoot markers by TCSA contradicts the weak perturbation of transcriptional hormone responses, so TCSA likely affects other processes that converge with CK signaling during regeneration.

**Figure 5:**
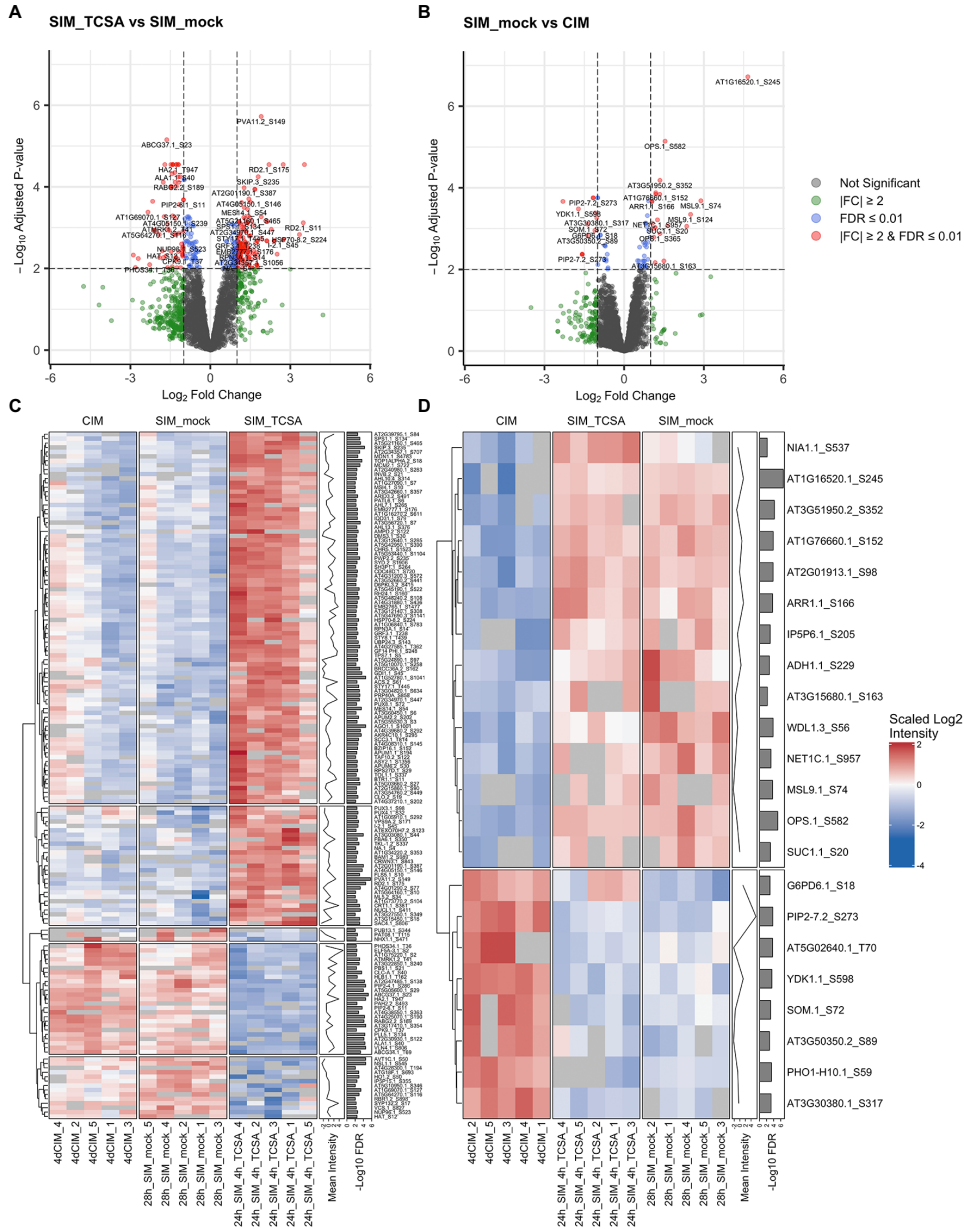
Phosphoproteome analysis of root explants on CIM and SIM following TCSA or mock treatment. (**A,B**) Volcano plots comparing phosphosite intensities after 28 h on SIM with 10 μM TCSA or mock treatment in the last 4 h (SIM_TCSA vs. SIM_mock), and after 28 h on control SIM or 4 days on CIM (SIM_mock vs. CIM). Gray dots represent non-significant values, green dots reflect absolute fold changes (FC) above 2, blue dots show phosphosites with an FDR below 0.01, and red dots indicate significant phosphosites (at FDR ≤0.01) with |FC| ≥2. (**C,D**) Heatmaps of scaled log_2_ intensities for top DRPs between root explants treated with TCSA or mock from 24 to 28 h on SIM (24h_SIM_4h_TCSA vs. 28h_SIM_mock; red dots in panel **A**), and after 24 h on SIM or after 4 days on CIM (28h_SIM_mock vs. 4dCIM; red dots in panel **B**). Clustering is based on Euclidean distances and annotation columns show mean log_2_ intensities (before scaling) and -log_10_ FDR values per row. In case of redundance only the most significant phosphosite per gene was retained.

### Phosphoproteome analysis uncovers TCSA-responsive phosphosites

Because TCSA is a putative kinase inhibitor and mutant analyses hint at multiple targets including CK signaling components and shoot meristem markers, a phosphoproteome analysis was conducted to uncover phosphorylation dynamics underlying TCSA-responsive shoot regeneration. To prioritize events parallel and downstream of CK perception, we focused on Ser/Thr/Tyr rather than His phosphoproteins and selected a long application window in terms of PTMs (4 h). Specifically, the phosphoproteome of root tissues was evaluated after TCSA or mock treatment from 24 to 28 h on SIM. As an additional control, we collected samples just before transfer to SIM. Total protein content was extracted, digested, and enriched for O-phosphopeptides prior to liquid chromatography (LC)-MS/MS analysis. Peptide identification and quantification were done in MaxQuant and the DEqMS package (based on limma) was used for statistical testing (Cox and Mann, 2008; Ritchie et al., 2015; Tyanova et al., 2016a; Zhu et al., 2020b). Comparing TCSA and mock-treated roots reveals 324 differentially regulated phosphosites (DRPs) at an FDR of 0.01, of which 202 are upregulated and 122 are downregulated upon TCSA treatment (Table 1; Figure 5A; Supplementary Data 1). The intensity of 205 of these DRPs increased or decreased at least two-fold (either implying that the site undergoes phosphorylation changes or that the (phospho) peptide abundance varied). The majority of DRPs concern S residues, while 40 are T residues. Of the 243 unique proteins identified, respectively 190, 37, 9, and 6 proteins harbor one, two, three or four DRPs. Additionally, the amino acid transporter AVT1C contains seven deregulated serine residues, six of which exhibit strong negative fold changes (Table 1). A heatmap of phosphosites (one per gene) with FDRs below 0.01 and absolute log_2_ fold changes (LFCs) above 1 highlights the discrepancy between CIM and control SIM on one hand and TCSA treatment on the other hand (Figure 5C). Hierarchical clustering roughly distinguishes five clusters, the two largest of which contain DRPs with low to moderate intensities on control SIM or CIM that are upregulated by TCSA, while three smaller clusters show moderate to high values for mock or CIM and downregulation after TCSA treatment. Intriguingly, many sites show opposite behavior when comparing CIM to mock or TCSA samples, suggesting that TCSA reverses phosphorylation trends required for regeneration. Based on statistical significance and fold changes, the most interesting candidate genes are *AGO1*, *RD2*, *AT1G52780*, *PVA11*, and *AVT1C* (FDR ≤ 10^−4^ and|LFC| ≥ 2). Other highly significant sites are located in ABCG37, PIP2;4/PIP2F, NSL1, CARK1, HA2, and VLN4 (Table 1). Three and 12 phosphosites are, respectively detected in TCSA or mock-treated samples only (Supplementary Table 1; Supplementary Data 1).

**Table 1:** Deregulated proteins containing at least one highly significant phosphosite (FDR ≤ 10^−4^) when comparing TCSA to mock SIM and/or mock SIM to CIM treatment (Supplementary Data 1).

Contrast	Protein	Site	P_i_	LFC	Mean	FDR	Gene ID	Description
TCSA vs. mock	PVA11.2	S149	1	1.91	2.75	1.89E-06	AT3G60600	Vesicle-associated protein 1–1	
		S2	1	−1.92	−2.34	1.48E-03			
	ABCG37.1	S23	1	−1.64	−0.50	7.02E-06	AT3G53480	ABC transporter G family member 37	
	AGO1.1	S1001	1	3.53	−0.94	2.84E-05	AT1G48410	Protein argonaute 1	
	AT1G52780.1	S1041	1	2.73	−2.15	2.84E-05	AT1G52780	PII, uridylyltransferase (DUF2921)	
	RD2.1	S175	1	2.20	−0.33	2.84E-05	AT2G21620	Responsive to desiccation 2	
		S11	1	3.49	−1.51	7.60E-04			
	AVT1C.1	S50	1	−1.71	−1.49	2.84E-05	AT2G39130	Amino acid transporter AVT1C	
		S126	2	−1.41	0.95	2.84E-05			
		S127	2	−1.41	0.95	2.84E-05			
		S87	1	−1.48	−1.10	9.81E-05			
		S132	2	−2.16	−0.85	2.26E-04			
		S126	1	−1.03	−0.26	1.23E-03			
		S14	1	1.04	0.57	2.31E-03			
		S92	1	−1.20	0.90	3.83E-03			
		S132	1	−0.70	−0.46	9.57E-03			
	NSL1.1	S545	1	−1.41	−2.52	2.84E-05	AT1G28380	MACPF domain-containing protein NSL1	
	CARK1.1	S354	2	−1.27	1.59	2.84E-05	AT3G17410	Receptor-like cytoplasmic kinase 1	
		T358	2	−1.27	1.59	2.84E-05			
	HA2.1	T947	1	−1.20	3.88	2.84E−05	AT4G30190	ATPase 2, plasma membrane-type	
		T942	1	−1.29	0.90	1.48E−03			
	VLN4.1	S806	1	−1.19	2.18	2.84E−05	AT4G30160	Villin-4	
		S787	1	−0.84	3.31	1.39E-03			
	ALA1.1	S40	1	−1.33	1.36	4.47E-05	AT5G04930	Phospholipid-transporting ATPase 1	
		S6	1	−0.77	0.64	6.00E-04			
		S20	1	−1.03	0.21	4.76E-03			
	PLL5.1	S134	1	−1.44	−0.76	4.80E-05	AT1G07630	Probable protein phosphatase 2C 4	
		S153	1	−1.27	−1.67	9.71E-05			
	SKIP.3	S235	1	1.79	−0.88	5.66E-05	AT1G77180	SNW/SKI-interacting protein	
		S235	2	0.94	4.41	3.13E-03			
		S243	2	0.94	4.41	3.13E-03			
	AT1G77180.1	S190	1	−1.13	0.77	5.66E-05	AT1G77180	Caldesmon-like protein	
		S315	1	−1.32	−1.06	7.72E-05			
		S274	1	−1.48	−0.33	9.98E-05			
	AT2G47485.1	S138	1	−1.21	0.25	5.73E-05	AT2G47485	Hypothetical protein	
	AT1G56140.1	S1003	1	−0.99	−1.22	6.67E-05	AT1G56140	LRR receptor-like Ser/Thr-protein kinase	
	CASP.1	S589	1	−0.97	1.69	6.67E-05	AT3G18480	Protein CASP	
	AT2G30930.1	S122	1	−1.77	−0.02	7.72E-05	AT2G30930	Expressed protein	
	RABG2.2	S189	1	−1.19	−0.75	7.72E-05	AT2G21880	Ras-related protein RABG2	
	JOX2.1	S29	1	−1.44	0.84	9.98E-05	AT5G05600	Jasmonate-induced oxygenase 2
TCSA vs. mock, (mock vs. CIM)	PIP2-4.1	S286	1	−1.48	2.63	2.84E-05	AT5G60660	Probable aquaporin PIP2-4
Mock vs. CIM	NAIP2.1	S245	1	4.66	1.30	1.90E-07	AT1G16520	NAI1 interacting protein
	OPS.1	S582	1	1.54	−0.99	7.19E-06	AT3G09070	Protein OCTOPUS
		S241	1	1.17	−1.29	9.35E-04		
		S365	1	1.35	−1.53	1.32E-03		
	AT3G51950.2	S352	1	1.35	−1.09	6.54E-05	AT3G51950	Zinc finger CCCH domain protein 46

Notably, 4 h of TCSA treatment has a stronger impact on phosphoproteome dynamics than 24 h incubation on cytokinin-rich SIM, as only 78 phosphosites are differentially regulated on control SIM versus CIM at FDR ≤ 0.01 (Table 1; Supplementary Data 1). Respectively 50 and 28 of these show increased and reduced levels on SIM, with 32 undergoing at least two-fold intensity changes (Figure 5B). These DRPs correspond to 58 unique proteins, 12 of which contain two or three phosphosites (and two of the three downregulated sites in the aquaporin PIP2;7/PIP3A can carry one or two phosphate groups). Respectively 68, 9, and 1 of the positions involve S, T, and Y residues. For DRPs with FDRs below 0.01 and LFCs above 1, a heatmap reveals similar trends on SIM with mock or TCSA treatment compared to CIM samples, and two clusters can be discerned (Figure 5D). The largest is made up of DRPs with increased phospholevels on SIM versus CIM, while the smaller one shows opposite behavior. Intriguingly, NAIP2.1 shows 25-fold higher intensity of phosphorylated S245 after 24 h on SIM and is required for the biogenesis of ER-derived vesicles (Wang et al., 2019b). Other top phosphosites are in OPS and AT3G51950 (FDR ≤ 10^−4^), MSL9 and AT5G02640 (|LFC| ≥ 2). Notable prior candidates are ARR1 (containing three upregulated S residues at positions 166, 168, and 190), QKY, and BAM1 (both harboring one upregulated serine). Although the affected serines in ARR1 are not implicated in the canonical His-Asp CK signaling phosphorelay, differential regulation of ARR1 on SIM vs. CIM but not TCSA vs. mock supports a role in shoot regeneration based on mutant analyses (Figures 3A,F,K,P) and confirms that it is not a direct TCSA target. Furthermore, 4 out of 13 plasma membrane (PM) integral proteins (PIPs) in *Arabidopsis* undergo reduced S/T phosphorylation on SIM (PIP2;7, PIP2;2, PIP2;6, and PIP2;4). PIP2;6 and PIP2;4 have an FDR ≤ 0.01 in both comparisons (TCSA vs. mock or control SIM vs. CIM treatment), along with BAM1, NIA1, EDR2, and AT4G38550. Finally, five phosphosites are found only after CIM incubation and two phosphosites are unique for mock SIM (Supplementary Table 1; Supplementary Data 1).

### Enriched functionalities among deregulated phosphoproteins

**Figure 6:**
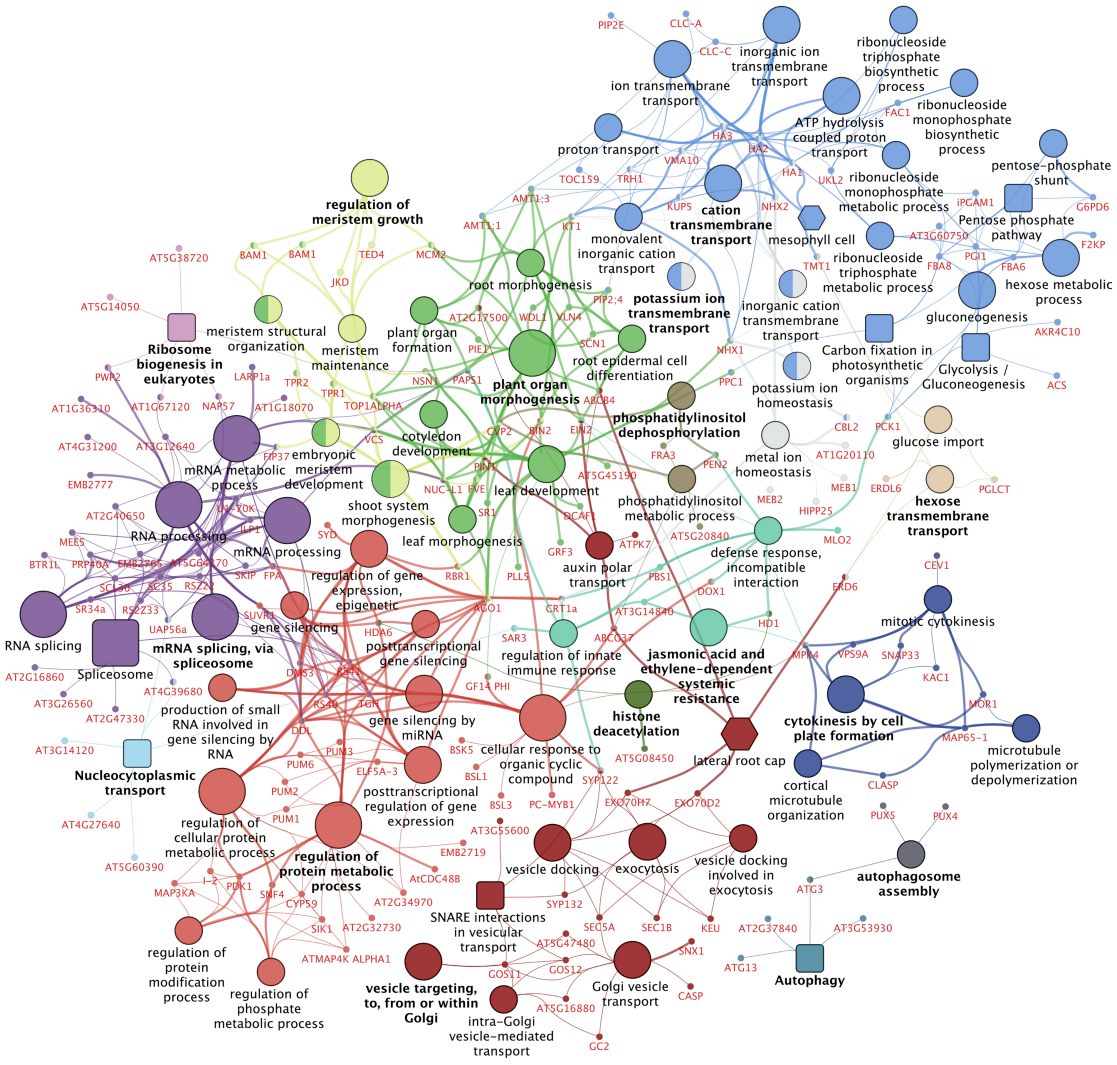
Gene set enrichment analysis on differentially regulated phosphoproteins after TCSA treatment. The network shows Gene Ontology (GO) Biological Process and Immune System Process terms [circular nodes (Ashburner et al., 2000; The Gene Ontology Consortium, 2014)], KEGG pathways [square nodes (Kanehisa and Goto, 2000)], Plant Ontology (PO) Anatomy—Plant Structure and PO Temporal—Plant Growth and Developmental Stage terms [hexagonal nodes (Avraham et al., 2008)] that are enriched among the 522 genes containing significantly deregulated phosphosites (FDR ≤ 0.05) between 4 h TCSA or mock treatment on SIM. Large nodes with black labels represent 75 functional terms with FDR ≤ 0.03, 5 ≤ GO tree interval level ≤ 15, and ≥ 3 associated genes, while small nodes with red labels depict the 174 input genes linked to these terms. Functional terms are connected based on the similarity of their associated genes (kappa score ≥ 0.3) and their size is proportional to the FDR. Colors reflect groups of related terms (≥50% overlap), and bold labels highlight the term with the lowest FDR per group. The graph was made with ClueGO and CluePedia in Cytoscape (Shannon et al., 2003; Bindea et al., 2009, 2013).

To obtain more insight into the molecular pathways and processes affected by TCSA, a gene set enrichment analysis (GSEA) was performed on all 522 genes that harbor significantly deregulated phosphosites between TCSA and mock treatment (at FDR ≤ 0.05). The results are presented in a functional network produced by the ClueGO plugin for Cytoscape (Shannon et al., 2003; Bindea et al., 2009, 2013), showing 75 enriched terms (alongside 174 input genes mapping to these terms), organized into 16 groups and connected based on gene overlap (Figure 6; Supplementary Figure 2). Overview terms per group were selected by significance. The most important clusters relate to protein modification (bright red; 11 terms and 38 genes), mRNA splicing (purple; 6 terms and 38 genes), Golgi vesicle transport (dark red; 9 terms and 22 genes), plant organ morphogenesis (bright green; 10 terms and 34 genes), cation transmembrane transport or gluconeogenesis (bright blue; 20 terms and 31 genes), and meristem growth (light green; 5 terms and 17 genes). The occurrence of terms such as meristem maintenance, embryonic meristem development, shoot system morphogenesis, leaf development, root morphogenesis, root epidermal cell differentiation, etc. corroborates that TCSA interferes with multiple (phospho) proteins related to *de novo* organogenesis. The top deregulated proteins (FDR ≤ 10^−3^ and|LFC| ≥ 1) in this group are VLN4, AGO1, PLL5, TOP1ALPHA, and MCM2, while other interesting members with respect to literature are BAM1, PIN1, EIN2, BIN2, TPR2, JKD, RBR1, and FIP37.

Enrichment of terms like cellular response to organic cyclic compound, regulation of protein modification process, and regulation of phosphate metabolic process further shows that TCSA influences post-translational control mechanisms. This involves I-2, GF14 PHI, PC-MYB1, ELF5A-3, DMS3, and SNF4. Less anticipated pathways include exocytosis, vesicle docking, and auxin polar transport (e.g., ABCG37, CASP, SYP132, and GSO11), inorganic ion transmembrane transport (e.g., HA2, CLC-A, and NHX1), RNA splicing, mRNA processing, and post-transcriptional gene silencing (e.g., AGO1, SKIP, PRP40A, PWP2, and AT2G34970). These imply extensive cellular deregulation by TCSA treatment. Moreover, carbon assimilation and energy metabolism are also affected. Noteworthy smaller groups are those involving defense responses, cytokinesis, histone deacetylation, and autophagy. When less stringent enrichment filters are applied, additional terms emerge (Supplementary Figure 2), such as signal transduction by protein phosphorylation (e.g., ATMAP4K ALPHA1, MAP3KA, MEKK1, SIK1), brassinosteroid-mediated signaling pathway (e.g., BIN2, BSK5, BSL1, BSL3, GF14 PHI), and cellular response to heat (e.g., BAG7, HSBP, LARP1a). Central hubs in the network are RS40&41, TGH, HA1-3, AGO1, VCS, and DDL (degree ≥10), while SYP122, MPK4, AGO1, NHX1, PAPS1, KT1, VCS, PCK1, and CRT1a form important connections between distant hubs (betweenness ≥ 3,000).

## Discussion

Consistent with the reported inhibition of cytokinin signaling by salicylanilides in periwinkle cells (Papon et al., 2003), TCSA impedes shoot regeneration from roots in *Arabidopsis thaliana*, which is most effective for short applications in the first 4 days of SIM incubation. The fold change of the effect was largest in Ws, while the absolute difference correlated with the regenerative potential of tested accessions. Mutant analyses showed that TCSA interferes directly or indirectly with AHKs and AHPs, of which AHK3, AHK4, AHP3, and AHP5 are prime candidates. Further biochemical evidence is needed to assess whether these interactions are direct. Inhibition of His autokinase activity by salicylanilides in bacterial two-component systems occurs *via* structural alteration and aggregation of the catalytic domain, supporting the notion that both AHKs and AHPs could be directly affected by TCSA, because both harbor a conserved His kinase domain (Stephenson et al., 2000). Although other HKs such as CKI1 and ETR1 have been linked to *de novo* organogenesis (Kakimoto, 1996; Chatfield and Raizada, 2008), it is unlikely that these are critical for inhibition of shoot regeneration by TCSA based on the genetic data and limited perturbance of TCSn activity by TCSA in WT. Repressing ARR1 functionality enhanced regeneration, whereas increased ARR1 activity reduced regeneration and TCSA susceptibility. Although ARR1 is a transcriptional activator in the CK signaling pathway (Sakai et al., 2000), it inhibits shoot regeneration through competition with ARR12 (Meng et al., 2017; Zhang et al., 2017; Liu et al., 2020). Because the latter induces *WUS*, an imbalance in active B-type ARR levels caused by TCSA might explain the observed effects. Notably, our phosphoproteome data also reveal deregulation of ARR1 S-phosphoproteins on SIM vs. CIM, but not in response to TCSA. Although serine phosphorylation of ARR1 has not been reported, CK prevents proteasomal degradation of this TF and serine phosphorylation of A-type ARRs enhances their stability (Kurepa et al., 2014b; Huang et al., 2018). As we find three upregulated phosphoserines in ARR1.1 upon CK treatment (S166, S168, and S190), it is possible that CK exploits similar mechanisms to mediate the activity of B-type ARRs. Reporter lines further reveal that TCSA application delays *WUS* expression and impairs *STM* induction on SIM, which is at least partly independent of CK signaling. Although Closantel® was reported to have similar specificity as TCSA (Papon et al., 2003), shoot regeneration from root explants is resistant to this inhibitor. This agrees with the lower potency of Closantel® in periwinkle and suggests that it might be degraded after prolonged exposure to light, metabolized, or poorly absorbed in *Arabidopsis* root cells. Additionally, our results show that rapamycin can enhance *de novo* shoot organogenesis, indicating that AtFKBP12 is not completely insensitive to the compound. This was also observed in cell suspension cultures, where many phosphopeptides underwent similar changes after treatment with rapamycin or the more potent TOR inhibitor AZD8055 (Van Leene et al., 2019), and under anaerobic conditions that partially restore AtFKBP12 functionality to retard growth in the presence of rapamycin (Deng et al., 2016). Accordingly, inconsistent rapamycin resistance in *Arabidopsis* has been attributed to variable endogenous FKBP12 content (Xiong and Sheen, 2012). As TOR is required for auxin responses, rapamycin could enhance shoot formation on SIM by tilting the balance of auxin-cytokinin antagonism in favor of CK signals.

A phosphoproteome analysis shows that besides inhibition of His phosphorylation, TCSA also has a strong impact on phosphorylation of Ser, Thr and Tyr, because 324 O-phosphosites are significantly deregulated after 4 h of TCSA treatment (at FDR ≤ 0.01; Supplementary Data 1). By contrast, only 78 DRPs are found when comparing explants kept on control SIM for 24 h vs. CIM. PIP2;4, PIP2;6, AT4G38550, BAM1, NIA1, and EDR2 contain DRPs that respond to both treatments, but only half of the targeted residues are conserved and all show similar trends when comparing TCSA and mock or SIM and CIM. Hence, the link with disrupted cytokinin signaling is difficult to assess. Černý et al. previously showed that many early cytokinin response phosphopeptides relate to protein regulation and gene expression (Cerny et al., 2011), which are also enriched ontologies in our data. Despite detecting only 29 phosphoproteins, they reported increased levels of phosphorylated COR47, which is downregulated by TCSA. A comparison of Ser/Thr/Tyr phosphorylation in WT and *ahk2 ahk3* mutants (with or without cytokinin) further identified nine proteins that also exhibit differential phospholevels after mock or TCSA treatment (e.g., PIP2;4, NET1C, and CDC48D), although most show conflicting trends (Dautel et al., 2016). Still, the number of unique phosphoproteins that respond to TCSA (243) is similar to the number of differentially phosphorylated proteins during protoplast regeneration in *Physcomitrium patens* (i.e., 300), and substantial overlap exists among enriched annotations (e.g., transcriptional regulation, transport, metabolism, cell division, and morphogenesis; Wang et al., 2014b). On the other hand, only nine phosphoproteins were deregulated during dedifferentiation of cotyledon cells in *Arabidopsis*, but this is likely due to the poor resolution and sensitivity of 2-DE gel assays (Chitteti and Peng, 2007). Although the latter study detected several 14–3-3-like proteins and such factors also appear in our phosphoproteome, none of the phosphosites overlap with our DRPs. Surprisingly, the majority of TCSA-responsive phosphosites exhibit increased intensities, despite its putative role as a His kinase inhibitor (Papon et al., 2003; Desikan et al., 2008). It is possible that these observations reflect indirect effects because sampling was done after 4 h and our analysis focused on Ser/Thr/Tyr residues. If and how elevated Ser/Thr/Tyr phospholevels contribute to impaired shoot regeneration depends on the role of deregulated phosphoproteins in organogenesis and the effect of phosphorylation on their activity. Interpretation of the results is further complicated because we cannot distinguish between differential phosphorylation or peptide abundance.

Twenty-four phosphopeptides are unique for a specific treatment group (Supplementary Table 1; Supplementary Data 1), but they are not linked to obvious candidates and could reflect negligible differences. Based on statistical significance, top TCSA targets are *AGO1*, *RD2*, *AT1G52780*, *PVA11*, and *AVT1C* (Table 1; Supplementary Data 1). ARGONAUTE (AGO) proteins mediate (post)transcriptional gene regulation through association with microRNAs (miRNAs) to form RNA-induced silencing complexes (RISC; Wilson and Doudna, 2013). We find that the serine residue at position 1,001 in AGO1.1 is more than 11-fold upregulated upon TCSA treatment. This phosphosite is also deregulated by H_2_O_2_ and was suggested to affect silencing activity because it is just downstream of the PIWI domain (Bentem et al., 2008). AGO1 is involved in various aspects of plant development and mutants show pleiotropic phenotypes (Kidner and Martienssen, 2005; Zhang and Zhang, 2012). Together with its close homolog AGO10/ZLL/PNH, AGO1 controls stem cell maintenance in the SAM by fine-tuning the activity of miR165/166, which target HD-ZIP III TFs (e.g., *PHB*, *PHV*, and *REV*) that interact with B-type ARRs to potentiate *WUS* expression (Zhang et al., 2017; Chang et al., 2020). While *AGO1* is ubiquitously expressed and promotes degradation of *HD-ZIP III* transcripts *via* miR165/166, AGO10 is confined to the SAM and the adaxial side of organ primordia to protect HD-ZIP III TFs by sequestering miR165/166 (Liu et al., 2009; Zhu et al., 2011; Zhang and Zhang, 2012; Zhou et al., 2015). Suppression of HD-ZIP III TFs by AGO1 further depends on miR168-directed autorepression and availability of SQN and HSP90 chaperones (Du et al., 2020). Notably, AGO1 was previously suggested to modulate *STM* levels (possibly involving *CUC1-2* targeting by miR164), and HD-ZIP III TFs can activate *STM* as well (Laufs et al., 2004; Kidner and Martienssen, 2005; Shi et al., 2016). Enhanced AGO1 function by phosphorylation might thus hamper shoot formation by repressing *HD-ZIP III* and downstream *WUS* (and/or *STM*) independent of CK (Zhang and Zhang, 2012; Chang et al., 2020). However, conflicting roles have been reported for the AGO10—miR165/166—HD-ZIP III module during *in vitro* shoot regeneration (Xue et al., 2017; Lardon and Geelen, 2020).

Phospholevels are also increased by TCSA at two positions in RD2.1, with over 11-fold changes for S11 and over four-fold changes for S175. RD2 is involved in dehydration and desiccation responses, but it has not been associated with organogenesis before. Next, AT1G52780 encodes a poorly characterized PII-uridylyltransferase (DUF2921) associated with the trans-Golgi network and S1041 in AT1G52780.1 shows more than six-fold higher intensity after exposure to TCSA (Groen et al., 2014). Uridylylation of PII signal transduction proteins mediates carbon and nitrogen sensing in the chloroplasts, but the link with regeneration is unclear (Hsieh et al., 1998; Chen et al., 2006). Vesicle-associated protein 1–1 (PVA11/VAP27-1) is required for endocytosis and autophagy through interaction with NET3C, the cytoskeleton, phosphatidylinositol-phosphate lipids, and clathrin at contact sites between the endoplasmic reticulum (ER) and the PM (Wang et al., 2014a, 2019a; Stefano et al., 2018). Misexpression causes pleiotropic defects, including aberrant root development (Wang et al., 2016). Phosphorylation is over three-fold increased or decreased at S149 and S2 in PVA11.2, respectively. Although proper endocytic trafficking is a prerequisite for *de novo* organogenesis (Dhonukshe et al., 2007; Kitakura et al., 2011; Marhavý et al., 2011; Liu et al., 2018), the effect of phosphorylation on PVA11 activity is unknown. The AVT1C protein contains seven deregulated S residues, six of which are downregulated with fold changes from two to four (S50, S87, S92, S126, S127, and S132; note that S126 and S132 can carry single or double modifications), while S14 is upregulated over two-fold. Another phosphosite in AVT1C (S91) is only detected on control SIM and CIM (Supplementary Table 1). Although little is known about this vacuolar amino acid transporter, *AVT1B* was predicted to be downregulated in shoots by high nitrogen-induced ARR4 (Heerah et al., 2021).

Other highly significant DRPs in response to TCSA are located in ABCG37 (a pleiotropic auxin transporter that exports indole-3-butyric acid from the root apex (Růžička et al., 2010)), PIP2;4 (an aquaporin with a DRP at S286 that is also targeted by SIRK1 and phosphorylated under salt stress (Hsu et al., 2009; Wu et al., 2013)), NSL1 [a perforin restraining SA-induced defense responses (Noutoshi et al., 2006)], CARK1 [a kinase that enhances drought resistance by phosphorylating ABA receptors (Zhang et al., 2018)], HA2 [a PM H^+^-ATPase required to establish the proton motive force (PMF; Haruta and Sussman, 2012)], and VLN4 [an actin filament bundling protein involved in root hair growth (Zhang et al., 2011)]. Phosphorylation of T947 in HA2 hyperactivates the enzyme by creating a binding site for regulatory 14–3-3 proteins (Fuglsang et al., 1999, 2007; Kinoshita and Shimazaki, 1999). This site is downregulated by TCSA (while the 14–3-3 proteins GRF3 and GF14 PHI show increased phospholevels), suggesting that a reduced PMF might hamper regeneration, consistent with the finding that *aha2* mutants show defective root growth under physiological stresses and *aha1 aha2* is embryo lethal (Haruta et al., 2010). Besides, cytokinin-responsive ARR1 and auxin promote cell wall acidification and loosening *via* H^+^-ATPases to induce elongation and differentiation (Hager et al., 1991; Fendrych et al., 2016; Pacifici et al., 2018). Finally, QKY is a prior candidate containing DRPs between mock SIM and CIM. We previously found that this transmembrane protein underlies natural variation in shoot regeneration and loss-of-function enhances callus growth under specific conditions (Lardon et al., 2020). QKY also interacts with the LRR-RLK SUB to control plasmodesmata conductivity during organ morphogenesis and mutation causes aberrant SAM structures (Fulton et al., 2009; Trehin et al., 2013; Vaddepalli et al., 2014). Here, we show that QKY.1 exhibits increased phospholevels at S258 after 24 h on SIM versus CIM, confirming a role in organogenesis.

For 39 of the 522 genes (7.5%) affected by TCSA (at FDR ≤ 0.05), GSEA revealed a link with organ morphogenesis. The most interesting candidates in this category based on literature are *BAM1*, *FIP37*, *PIN1*, *RBR1*, *TOP1ALPHA*, *AGO1*, *BIN2*, *DCAF1*, *EIN2*, *JKD*, *MCM2*, *NSN1*, *PAPS1*, *PLL5*, and *TPR2*. BAM1 and PLL5 are involved in SAM patterning, as BAM1 antagonizes CLV1 and promotes *WUS* expression *via* ligand competition and CLE40 signaling (DeYoung and Clark, 2008; Somssich et al., 2016; Schlegel et al., 2021). Although PLL5 was only attributed a role in leaf development, it is a homolog of POL, acting downstream of CLV1 in the repression of *WUS* by CLV3 (Song and Clark, 2005; Song et al., 2006). Accordingly, BAM1 and PLL5 show contrasting phosphorylation trends in response to TCSA, but how this modulates their activity remains to be elucidated. FIP37 controls shoot stem cell fate by confining the expression of *WUS* and *STM* through destabilizing N^6^-methyladenosine modification of mRNA, and loss-of-function causes SAM overproliferation (Shen et al., 2016). TOP1ALPHA/FAS5 (Graf et al., 2010; Liu et al., 2014; Albert et al., 2015; Zhang et al., 2016), RBR1 (Wildwater et al., 2005; Perilli et al., 2013; Ötvös et al., 2021), NSN1 (Wang et al., 2011, 2012), JKD (Welch et al., 2007; Bustillo-Avendaño et al., 2018), and MCM2 (Ni et al., 2009; Huang et al., 2019a) also mediate meristem maintenance and organ patterning at the shoot or root apex, but none of the DRPs were previously reported. Next, PIN1, PILS5, ABCB4, and ABCG37 play a role in (polar) auxin transport (Růžička et al., 2010; Barbez et al., 2012; Cho et al., 2012), which is pivotal to establish hormone gradients in organ primordia (Motte et al., 2014b; Lardon and Geelen, 2020). Phosphorylation by PID/WAG, D6PK, PAX, and MAP kinases controls directional auxin flow *via* activity modulation, polarity switches and re-localization of PINs (Tan et al., 2021). For instance, S337 in the cytoplasmic loop of PIN1 is phosphorylated by the MKK7—MPK3/6 cascade, causing a basal-to-apical shift during shoot branching (Jia et al., 2016; Barbosa et al., 2018). This phosphosite is upregulated by TCSA, potentially impeding *de novo* SAM formation by diminishing auxin maxima. MPK3/6 are also downstream of YDA, which is inhibited by BIN2-mediated phosphorylation (Kim et al., 2012). As BIN2 harbors a downregulated tyrosine (Y200) and autophosphorylation of this site is positively correlated with kinase activity (Li et al., 2020), BIN2 could act upstream of differential PIN phosphorylation in TCSA responses. Moreover, BIN2 is involved in auxin signaling and enhances induction of *LBD16* and *LBD29* by ARF7 and ARF19 during lateral rooting, which is also required for callus formation (Vert et al., 2008; Cho et al., 2013; Lee and Seo, 2017; Lardon and Geelen, 2020). On the other hand, the MPK3/6 module normally promotes embryonic patterning (Neu et al., 2019), and BIN2 suppresses BR signaling by phosphorylation-dependent degradation of BZR1, which is linked to reduced proliferation and regeneration (He et al., 2002; Cheon et al., 2010). Hence, the contribution of BIN2 to impaired shoot formation by TCSA is ambiguous.

BRs also control the formation of organ boundaries *via* CUCs (Gendron et al., 2012), and additional BR signaling factors containing TCSA-responsive DRPs are BSL1 and BSL3 (Kim et al., 2016), BSK5 (Li et al., 2012), GF14 PHI (interacting with BZR1 [Ryu et al., 2007)], and TPR2 [a homolog of the TOPLESS corepressor involved in auxin and BR responses, as well as SAM specification (Long et al., 2006; Espinosa-Ruiz et al., 2017; Martin-Arevalillo et al., 2017)]. Interestingly, BR-induced gene expression is modulated *via* selective autophagy of BES1 (requiring phosphorylation of DSK2 by BIN2; Nolan et al., 2017) and TCSA affects phosphosites in autophagy-related proteins (e.g., ATG1B, ATG1C, ATG3, ATG13A, and ATG18F). Moreover, ATEH1 and ATG13A phosphopeptides are not detected after TCSA treatment (Supplementary Table 1) and ATEH1 interacts with PVA11/VAP27-1 (containing two DRPs) at ER-PM contact sites to mediate endocytosis and autophagosome biogenesis (Wang et al., 2019a). Intriguingly, autophagy is required for proteome adjustments during hormone-induced reprogramming of somatic cells to pluripotent stem cells and subsequent redifferentiation (Rodriguez et al., 2020), providing an additional mechanism for TCSA-impaired shoot organogenesis. Besides, ATG8 proteins show interplay with cytokinin-regulated root architecture (Slavikova et al., 2008) and numerous genes are transcriptionally coregulated in *atg*, *ahk2 ahk3 ahk4*, and *arr1 arr10 arr12* mutants (Masclaux-Daubresse et al., 2014). The GSEA network also contains several exocyst subunits (e.g., EXO70D2, EXO70H7, SEC1B, and SEC5A), which promote autophagic degradation of A-type ARRs upon phosphorylation of a conserved D residue in the receiver domain to tweak CK sensitivity in response to carbon starvation (Acheampong et al., 2020). Whether this could also regulate B-type ARRs, such as ARR1, remains to be investigated.

In summary, the salicylanilide TCSA impairs shoot regeneration from roots when applied during the first 4 days of SIM incubation. Genetic analyses suggest that this is at least partially caused by interference with histidine kinases and phosphotransfer proteins involved in cytokinin signal transduction, such as AHK3, AHK4, AHP3, and AHP5. Phosphoproteomics further reveals profound deregulation of Ser/Thr/Tyr phosphorylation, which affects factors linked to protein modification, transcriptional regulation, vesicle trafficking, organ morphogenesis, and cation transport. Further research on TCSA-responsive phosphoproteins such as AGO1, BAM1, PLL5, PIN1, and BIN2, will determine whether these are direct targets or act downstream of the CK shoot induction pathway.

## Data availability statement

The raw data generated in this study can be found in the PRIDE repository with identifier PXD030754. Processed results of the phosphoproteome analysis are available in Supplementary Data 1.

## Author contributions

RL designed and performed experiments, analyzed and visualized data, and wrote the manuscript. HT and BC helped with experiments. XX and LV assisted in data analysis. MP provided resources. SV edited the manuscript. DG and IDS conceptualized research, supervised the project, and edited the manuscript. All authors contributed to the article and approved the submitted version.

## Funding

This work was supported by the Research Foundation Flanders (FWO; application numbers 1S48517N and G094619N).

## Conflict of interest

The authors declare that the research was conducted in the absence of any commercial or financial relationships that could be construed as a potential conflict of interest.

## Publisher’s note

All claims expressed in this article are solely those of the authors and do not necessarily represent those of their affiliated organizations, or those of the publisher, the editors and the reviewers. Any product that may be evaluated in this article, or claim that may be made by its manufacturer, is not guaranteed or endorsed by the publisher.
