# Supplementary material for: Histidine kinase inhibitors impair shoot regeneration in Arabidopsis thaliana via cytokinin signaling and SAM patterning determinants
Source: Front Plant Sci. 2022 Sep 8;13:894208. doi: 10.3389/fpls.2022.894208 (PMC9847488; doi:10.3389/fpls.2022.894208)
Supplement: Supplementary file 2 [file Data_Sheet_2.docx]

Supplementary Material

# Supplementary Data

**Supplementary Data 1.** Excel file containing differentially regulated phosphosites (FDR ≤ 0.01) between TCSA and mock treatment or control SIM and CIM treatment, as well as uniquely detected phosphosites in each treatment group.

**Supplementary Data 2.** XML file with parameter settings used for (phospho)protein identification and quantification in MaxQuant (version 1.6.11.0).

**Supplementary Code.** R code used for statistical analysis of phosphoproteome data with DEqMS.

# Supplementary Figures and Tables

## Supplementary Figures


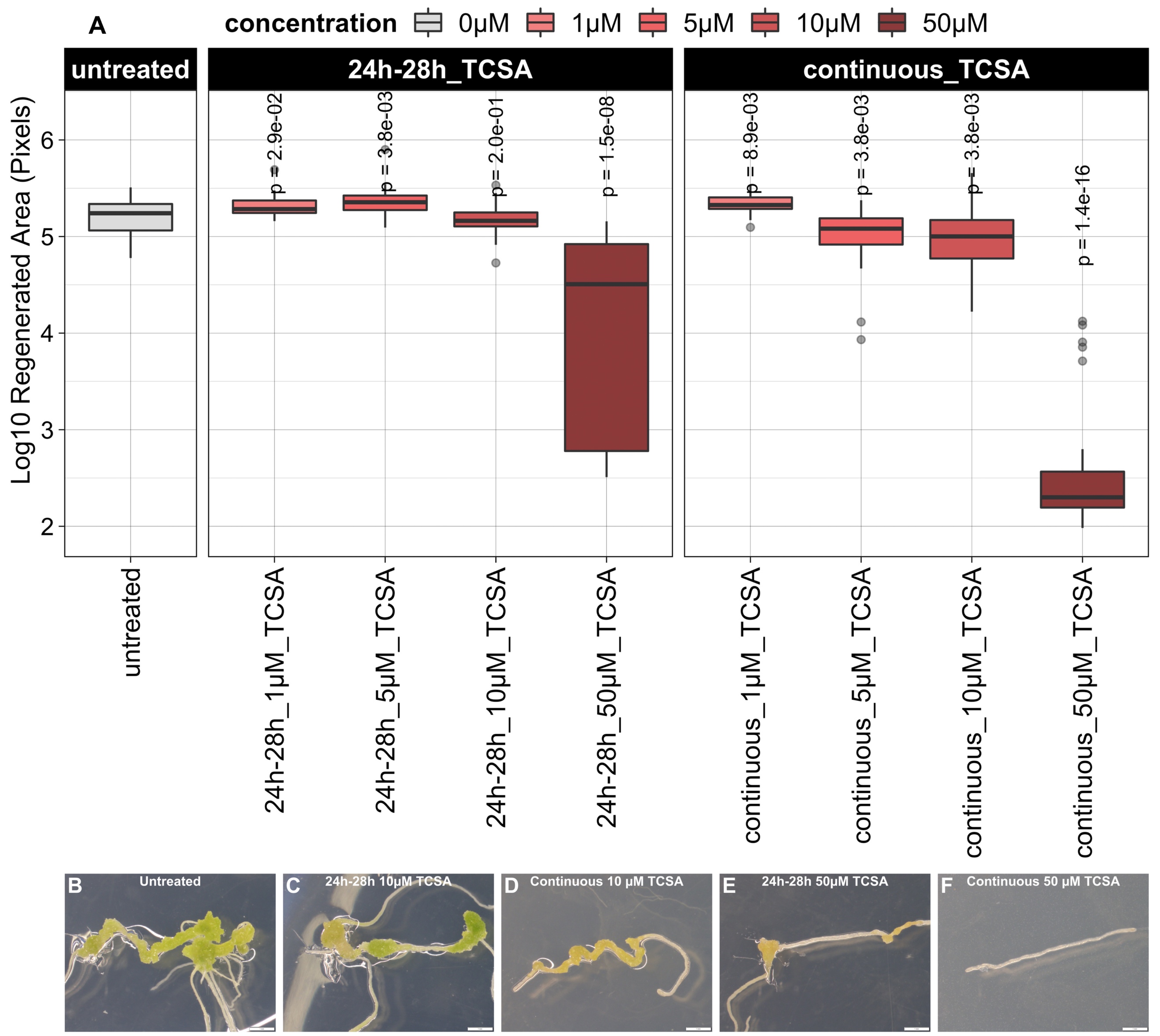


**Supplementary Figure 1. Effect of different TCSA concentrations on shoot regeneration from roots in *Arabidopsis thaliana* ecotype Ws. (A)** Boxplot showing the log_10_-transformed area of regenerated green tissue (in pixels) after supplementation of SIM with 1, 5, 10 or 50 µM TCSA from 24 h to 28 h or for the entire 21-day incubation period. For each treatment, ~30 individual explants were analysed. A Kruskal Wallis test revealed significant global differences (pKW = 2.6e-33) and FDR-adjusted p-values reflect post-hoc Wilcoxon tests relative to the untreated control. **(B)-(F)** Representative images of Ws root explants after 21 d on control SIM (**B**) and treatment with 10 µM (**C-D**) or 50 µM (**E-F**) TCSA from 24 h to 28 h (**C** and **E**) or for 21 days (**D** and **F**). Pictures were taken using binoculars (6.3x) and white scale bars represent 1 mm.


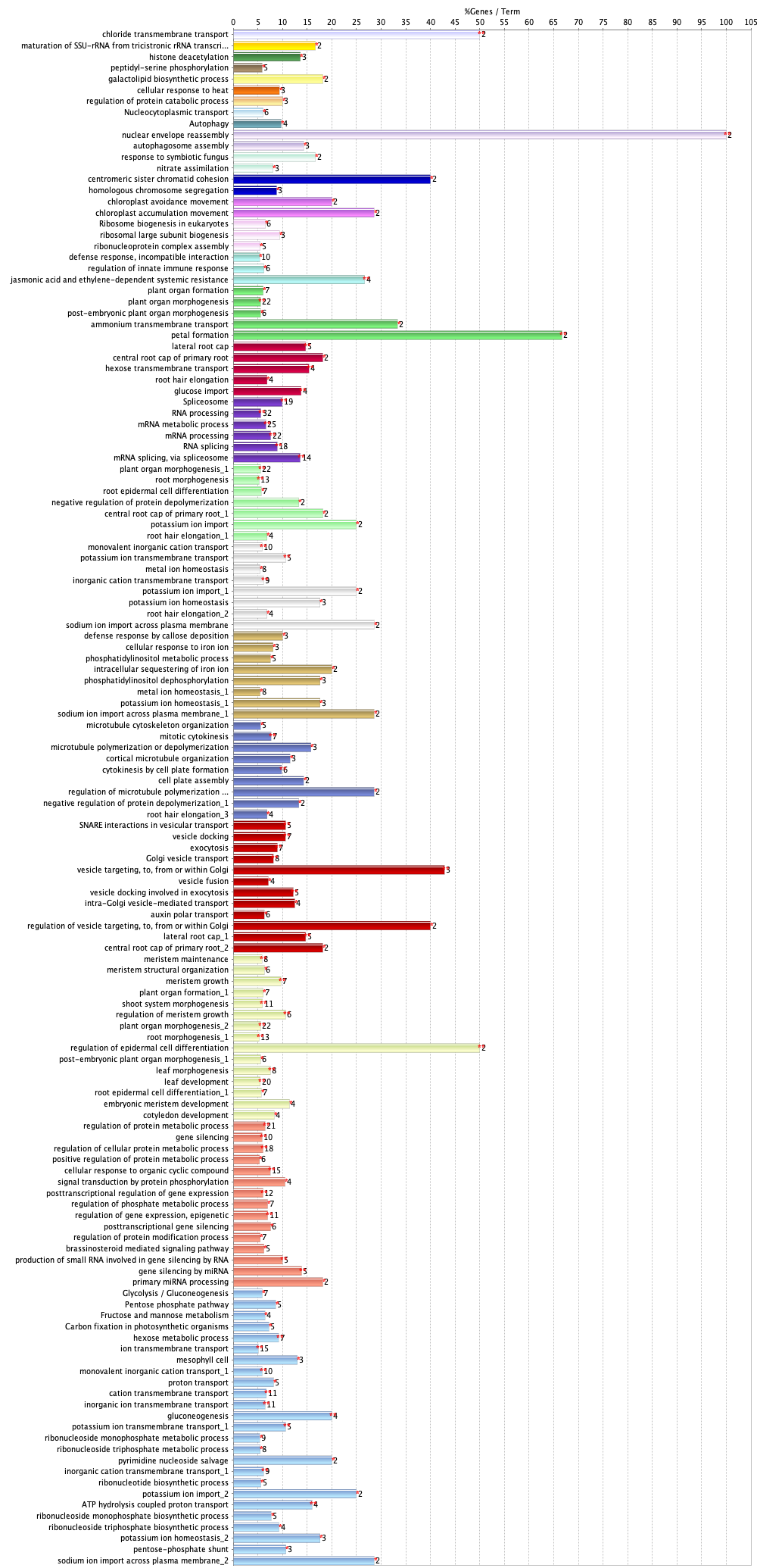


**Supplementary Figure 2. Low stringency GSEA on differentially regulated phosphoproteins by TCSA.** Bars show the percentage of associated genes for 115 enriched functional terms (with FDR ≤ 0.05, 5 ≤ GO tree interval level ≤ 15, ≥ 2 associated genes, and ≥ 5 % associated genes) among 522 genes containing significant DRPs between TCSA and mock treatment on SIM (FDR ≤ 0.05). Colours represent 26 groups; labels indicate the number of genes per term and asterisks (red) reflect FDRs from a right-sided hypergeometric test (* = p ≤ 0.05; ** = p ≤ 0.01).

## Supplementary Tables

**Supplementary Table 1.** List of unique phosphosites in each comparison (i.e., phosphosites that were detected in all five replicates of one condition but none of the other treatment group; Supplementary Data 1).

| **Contrast** | **Protein** | **Site** | **P_i_** | **N_TCSA_** | **N_mock_** | **N_CIM_** | **Description** |
| --- | --- | --- | --- | --- | --- | --- | --- |
| TCSA vs. mock | IREH1.1 | S219 | 1 | 5 | 0 | 0 | Probable serine/threonine protein kinase |
|  | RS31.1 | S130 | 1 | 5 | 0 | 2 | Serine/arginine-rich splicing factor |
|  | DEK4.1 | S113 | 1 | 5 | 0 | 2 | DEK domain-containing chromatin-associated protein 4 |
|  | AIR9.1 | S213 | 1 | 0 | 5 | 3 | 187-kDa microtubule-associated protein |
|  | BTZ2.1 | S52 | 1 | 0 | 5 | 4 | CASC3/Barentsz eIF4AIII binding protein |
|  | ATEH1.1 | S878 | 1 | 0 | 5 | 5 | Calcium-binding EF hand protein |
|  | AT5G45510.2 | T293 | 1 | 0 | 5 | 5 | Probable disease resistance protein |
|  | AT3G48450.1 | S53 | 1 | 0 | 5 | 5 | RPM1-interacting protein 4 (RIN4) protein |
|  | AVT1C.1 | S91 | 2 | 0 | 5 | 5 | Amino acid transporter AVT1C |
|  | AT4G38550.1 | S153 | 1 | 0 | 5 | 5 | Phospholipase-like protein (PEARLI 4) |
|  | ATG13A.1 | S337 | 2 | 0 | 5 | 5 | Autophagy-related protein 13a |
|  |  | S341 | 2 | 0 | 5 | 5 |  |
|  | GIR1.1 | S15 | 1 | 0 | 5 | 5 | Protein GL2-INTERACTING REPRESSOR 1 |
|  | AT3G09850.1 | S187 | 1 | 0 | 5 | 5 | D111/G-patch domain-containing protein |
|  | TULP10.1 | S50 | 1 | 0 | 5 | 5 | Tubby-like F-box protein 10 |
| Mock vs. CIM | RPS3AA.1 | S236 | 1 | 2 | 5 | 0 | 40S ribosomal protein S3a-1 |
|  | AT1G22060.1 | S261 | 1 | 4 | 5 | 0 | Sporulation-specific protein |
|  | EIF5B1.1 | S41 | 1 | 0 | 0 | 5 | Eukaryotic translation initiation factor 5B |
|  | STY17.1 | S146 | 1 | 0 | 0 | 5 | Serine/threonine-protein kinase STY17 |
|  | MGO3.1 | S259 | 1 | 0 | 0 | 5 | Uncharacterized protein |
|  | TIF3F1.1 | S14 | 1 | 2 | 0 | 5 | Eukaryotic translation initiation factor 3F |
|  | APD7.1 | S356 | 1 | 4 | 0 | 5 | Probable protein phosphatase 2C 67 |
| TCSA vs. CIM | NAIP2.1 | S191 | 1 | 5 | 1 | 0 | NAI1 interacting protein |
|  | RGGA.2 | S225 | 1 | 5 | 1 | 0 | RGG repeats nuclear RNA binding protein A |
